# Side‐On Coordination in Molecular Alkaline Earth Metal Hypofluorites FM(η^2^‐OF) (M = Mg, Ca, Sr, and Ba)

**DOI:** 10.1002/anie.202507151

**Published:** 2025-07-16

**Authors:** Xiya Xia, Robert Medel, Sebastian Riedel

**Affiliations:** ^1^ Institut für Chemie und Biochemie – Anorganische Chemie Freie Universität Berlin Fabeckstraße 34/36 14195 Berlin Germany

**Keywords:** Alkaline earth metals, IR spectroscopy, Non‐VSEPR structure, Side‐on coordination

## Abstract

Side‐on coordinated molecular hypofluorites FM(η^2^‐OF) (M = Mg, Ca, Sr, and Ba) were prepared by co‐deposition of laser‐ablated alkaline earth metal atoms with diluted OF_2_ in cryogenic matrices. The structures parallel the non‐VESPR structures of alkaline earth metal difluorides and dihydrides, such that FMg(η^2^‐OF) is planar, FCa(η^2^‐OF) is planar but with a shallow bending‐potential curve, while FSr(η^2^‐OF) and FBa(η^2^‐OF) are non‐planar. Bonding analyses show that the interactions between the metal center M^2+^ and the negatively charged ligands F^−^ and OF^−^ are mainly electrostatic. Moreover, for FMg(η^2^‐OF), donation of electron density from OF^−^ to empty s and p orbitals of magnesium is involved. However, for the heavier alkaline earth metal compounds, the donation to empty d orbitals of the metal center becomes dominant in orbital interactions, which contributes to their non‐planar structures.

## Graphical abstract

Side‐on coordinated alkaline earth metal hypofluorites F′M(η^2^‐OF) (M = Mg, Ca, Sr, and Ba) were prepared through the reactions of laser‐ablated metal atoms with OF_2_ in argon matrices. The species feature a fluorido F′^−^ and a side‐on coordinated η^2^‐OF^−^ ligand with the M─F─O─F′ dihedral angle increasing from calcium to barium, which parallel the non‐VESPR structures of alkaline earth metal difluorides.

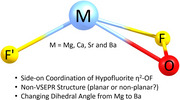

## Introduction

Molecular ternary metal oxyfluorides have attracted little attention so far, compared to binary metal oxides and fluorides. Mercury hypofluorite FHg(η^1^‐OF) was observed in the reaction with OF_2_.^[^ ^1^ ^]^ The group 12 metal mercury is considered as a post‐transition metal. It has some similar characteristics to alkaline earth metals^[^ ^2^ ^]^ because it also has a filled s orbital as its outer shell and its fully occupied d orbitals do not usually participate in chemical reactions. One known exception is the mercury(IV)fluoride HgF_4_.^[^ ^3^ ^]^ As for traditional transition metals, whose d orbitals are usually involved in chemical bonding, nearly all 3d transition metal (Sc–Cu) oxyfluorides have been investigated.^[^ ^4^, ^5^, ^6^, ^7^, ^8^, ^9^ ^]^ In addition to molecular OMF, the products OMF_2_ have been detected for these metals, all possessing *C*_2v_ symmetry, with a terminal oxygen atom, in part with oxyl character. For some metals, the slightly bent FM(η^1^‐OF) hypofluorite products, characterized by the stretching frequency of the O─F group, have been observed as well.^[^ ^8^, ^9^ ^]^

The monomolecular alkaline earth metal oxyfluorides were relatively rarely investigated. Recently, in our lab, an unusual beryllium oxyfluoride OBeF_2_ was observed in the reaction of laser‐ablated Be and OF_2_ in cryogenic matrices. This molecule can be described as a triplet oxygen atom stabilized by a relatively weak donor‐acceptor interaction with BeF_2_. Apart from that, OBeF, with oxyl radical character, was also observed in the same reaction.^[^ ^10^ ^]^ In 1971, Andrews and Raymond reported infrared‐spectroscopic studies of matrix reactions of OF_2_ with Li, Na, K, and Mg atoms.^[^ ^11^ ^]^ They observed some metal‐dependent bands. However, many bands could not be assigned due to a lack of supporting computational methods at that time. In particular, no magnesium oxyfluoride was identified.

Moreover, even within the group 2 elements, their chemical behaviors are considered different. The heavy alkaline earth metals Ba–Ca have low electronegativities of 0.89–1.00, followed by magnesium with 1.31, while beryllium has an electronegativity of 1.57 according to the Pauling scale.^[^ ^12^ ^]^ Thus, the heavy alkaline earth metal compounds are more ionic and the beryllium compounds often have a covalent character. However, a simple ionic model failed to explain the bent structures of some heavy alkaline earth metal dihalides in the gas phase.^[^ ^13^, ^14^ ^]^ Later, Kaupp et al. explained that core‐polarization and d orbital participation have a significant effect on bending.^[^ ^15^ ^]^ Due to their energetically accessible empty d orbitals, heavy alkaline earth metals can even exhibit some transition‐metal character, such as forming the eight‐coordinate carbonyl complexes.^[^ ^16^ ^]^ Therefore, it has been suggested that the heavy alkaline earth metals should be regarded as transition metals rather than main‐group elements,^[^ ^17^ ^]^ which encouraged us to investigate alkaline earth metal oxyfluorides further.

In the present work, we report the experimental observation of molecular alkaline earth metal hypofluorites, side‐on FM(η^2^‐OF) (M = Mg, Ca, Sr, and Ba), under cryogenic conditions in solid argon matrices. They are characterized by FTIR spectroscopy, quantum‐chemical calculations, and ^16/18^OF_2_ isotopic substitution experiments. The nature of the bonding is analyzed using natural bond orbital analysis (NBO) and extended transition state‐natural orbitals for chemical valence (ETS‐NOCV) analysis.

## Results and Discussion

According to calculations at both B3LYP‐D3 and CCSD(T) levels of theory, the direct insertion of alkaline earth metal atoms into the O─F bond of OF_2_, yielding FMOF, is highly exothermic in the order of 700 kJ mol^−1^ (detailed numbers are given in Scheme S1 in the Supporting Information). Its dissociation to OMF and F is highly endothermic (Scheme S2). For each metal, except for magnesium, two singlet isomers, one with side‐on and another one with end‐on coordination of the OF ligand, could be structurally optimized.

**Figure 1:**
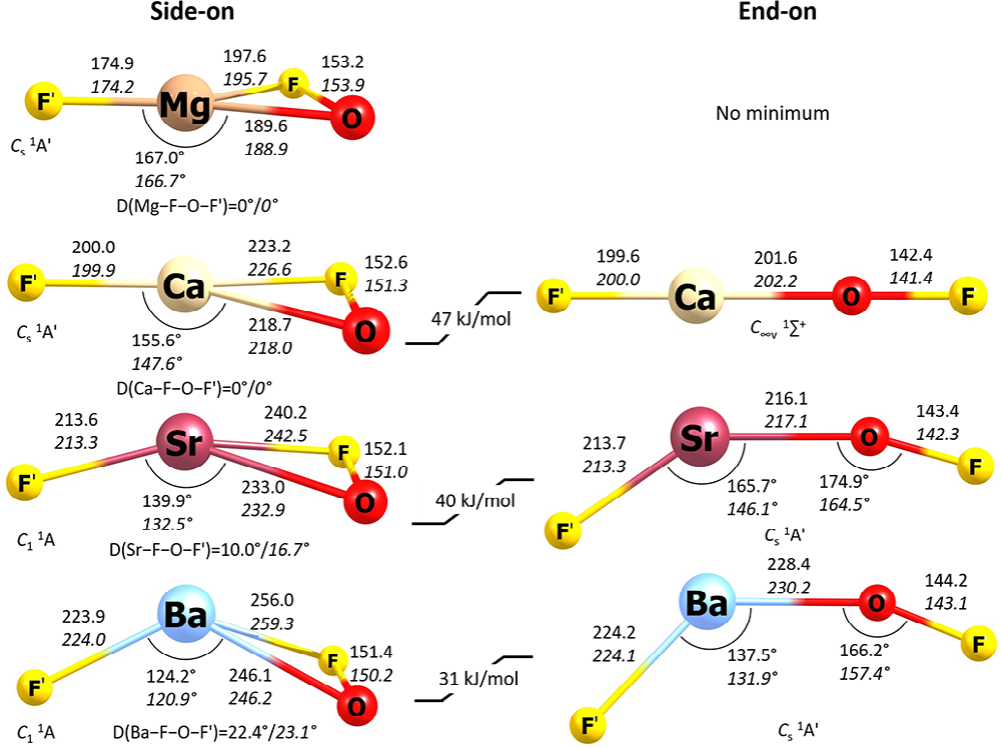
Optimized minimum structures of F′MOF (M = Mg, Ca, Sr, Ba) with a side‐on coordinated η^2^‐OF ligand (left) or an end‐on coordinated η^1^‐OF ligand (right). F′Mg(η^1^‐OF) is a second‐order transition state (not shown). Distances are given in pm. Values in normal font: B3LYP‐D3/def2‐TZVP, in *italic font*: CCSD(T)/aug‐cc‐pwCVTZ‐(PP). Energy differences between the side‐on and end‐on structures in kJ mol^−1^ were calculated at the CCSD(T)/aug‐cc‐pwCVTZ‐(PP) level. Side‐on structures are energetically lower than the end‐on structures. All energies were corrected by zero‐point energy.

For magnesium only the side‐on isomer is a minimum structure, whereas the linear end‐on structure has two imaginary frequencies (Tables S4, S16, and S19). The optimized structures of side‐on and end‐on F′MOF are shown in Figure 1 with the label F′ being used for the fluorido ligand. Side‐on F′Mg(η^2^‐OF) and F′Ca(η^2^‐OF) are planar and show *C*_s_ symmetry, while side‐on F′Sr(η^2^‐OF) and F′Ba(η^2^‐OF) are only optimized to show *C*_1_ symmetry. The dihedral angle M─F─O─F′ quantifies the deviation from planarity. It is zero, when all four atoms are in the same plane, but increases from Ca to Ba, resembling the decrease of the X─M─X angle in alkaline earth metal dihalides.^[^ ^14^ ^]^

For F′CaOF, F′SrOF, and F′BaOF, the side‐on structures are about 40 kJ mol^−1^ lower than the end‐on structures. However, at the CCSD(T) level, the quantum‐chemical treatment of the metal's core and semi‐core electrons significantly influences the energetic difference between side‐on and end‐on structures. Unfreezing the core electrons in combination with using dedicated basis sets for evaluating core‐valence correlation is crucial to achieve the correct energy value (for details see SI). The commonly used frozen core approximation can, however, invert the energetic order.

**Figure 2:**
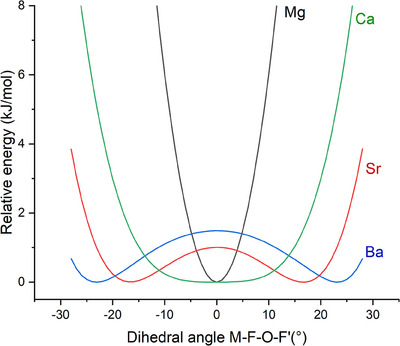
Scans of the electronic energy in dependence on the dihedral angle M─F─O─F′ for F′M(η^2^‐OF) (M = Mg, Ca, Sr, and Ba) at the B3LYP‐D3/def2‐TZVP level.

When scanning the M─F─O─F′ dihedral angle of the side‐on isomers, F′Mg(η^2^‐OF) has a sharp energetic minimum at 0°. F′Ca(η^2^‐OF) is found to be planar but with a very flat bending potential, while F′Sr(η^2^‐OF) and particularly F′Ba(η^2^‐OF) are significantly non‐planar (Figure 2). The energy barriers to planarity for F′Sr(η^2^‐OF) and F′Ba(η^2^‐OF) are ca. 0.7 (1.0) and 1.1 (1.5) kJ mol^−1^, respectively, with (without) harmonic zero‐point energy correction.

Similarly, the end‐on isomer of F′CaOF shows a linear structure, while end‐on F′SrOF and F′BaOF have *C*_s_ symmetry with the F′─M─O and M─O─F angles decreasing from Ca to Ba. This phenomenon of changing geometry down the group is also observed for other small heavy‐alkaline‐earth‐metal molecules, such as dihydrides and dihalides. Possible reasons are core‐polarization and d orbital participation.^[^ ^15^, ^18^ ^]^

The calculated O─F stretching wavenumbers, shown in Table 1, are considerably different between the side‐on and end‐on structures, and are not strongly influenced by the quantum‐chemical treatment of the metal center. Side‐on F′M(η^2^‐OF) should have an O─F vibration at about 800 cm^−1^, with a ^16/18^O isotopic shift of ca. 23–24 cm^−1^. Down the group, both the wavenumber and the shift increase. In contrast, the O─F stretching wavenumber of the end‐on structures would be expected to appear at around 1000 cm^−1^ with a corresponding ^16/18^O isotopic shift of 34–39 cm^−1^. Moreover, they display a different trend from Ca to Ba, where the O─F vibration and ^16/18^O shift of the end‐on F′M(η^1^‐OF) decrease from Ca to Ba. Thus, the side‐on and end‐on structures should be easily distinguishable by their O─F vibrational wavenumbers in the experiments.

**Table 1:** Calculated O─F vibrational wavenumbers ν(O─F) in cm^−1^, and ^16/18^O isotopic shifts Δν in cm^−1^ for side‐on and end‐on F′MOF (M = Ca, Sr, Ba) species.

	B3LYP‐D3^a)^		CCSD(T)^b)^	
ν(O─F)	ν(^16^O)	∆ν(^16/18^O)	ν(^16^O)	∆ν(^16/18^O)
F′Ca(η^1^‐OF)	1077.9	−39.6	1048.1	−39.5
F′Sr(η^1^‐OF)	1037.7	−37.0	1003.0	−37.0
F′Ba(η^1^‐OF)	999.6	−34.6	963.5	−34.5
F′Ca(η^2^‐OF)	808.1	−23.7	785.8	−23.2
F′Sr(η^2^‐OF)	811.8	−23.9	790.1	−23.5
F′Ba(η^2^‐OF)	826.0	−24.4	803.9	−23.8

^a)^ B3LYP‐D3/def2‐TZVP.

^b)^ CCSD(T)/awCVTZ‐PP without frozen core of metals.

A corresponding *C*_2v_ structure of the triplet OMF_2_, which is the global minimum for M = Be,^[^ ^10^ ^]^ is not a minimum for any of the heavier alkali earth metals. However, at the CCSD(T) level, a triplet minimum can be found for Mg–Sr, while triplet OBaF_2_ has one imaginary frequency. They are energetically higher than the side‐on singlet structures by 2.8 kJ mol^−1^ for Mg, 22.1 kJ mol^−1^ for Ca, 23.3 kJ mol^−1^ for Sr, and 24.9 kJ mol^−1^ for Ba (Table S2) and do not feature an O─F vibration. Complete calculation data are shown in Tables S6–S14 and S21–S29.

**Figure 3:**
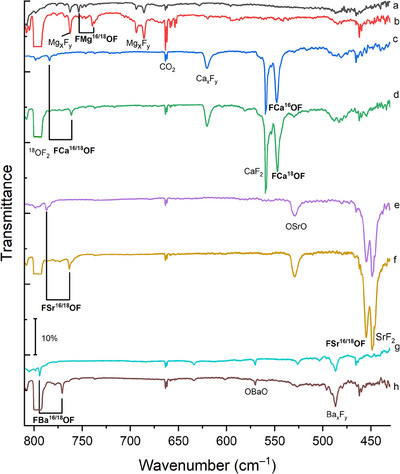
FTIR spectra of argon matrices after co‐deposition of a) Mg with 0.5% ^16^OF_2_ at 10 K, b) Mg with 0.5% ^18^OF_2_ at 10 K, c) Ca with 0.5% ^16^OF_2_ at 10 K, d) Ca with 0.5% ^18^OF_2_ at 10 K, e) Sr with 0.5% ^16^OF_2_ at 10 K, f) Sr with 0.5% ^18^OF_2_ at 10 K, g) Ba with 0.5% ^16^OF_2_ at 10 K, h) Ba with 0.5% ^18^OF_2_ at 10 K. Intense bands were partly truncated for illustration purposes.

The infrared spectra from co‐deposition of laser‐ablated metal atoms with 0.5 % ^16^OF_2_ or ^18^OF_2_ in argon at 10 K are shown in Figure 3. Metal fluoride bands are assigned based on experiments of metals with fluorine gas (Figure S1). Some metal oxides were also observed,^[^ ^19^ ^]^ due to the reactive nature of the alkaline earth metals with oxygen while handling the targets.

The IR spectra obtained after co‐deposition of laser‐ablated magnesium with OF_2_ are similar to those Andrews and Raymond reported in 1971.^[^ ^11^ ^]^ In their work, bands at 753.7, 746.5, and 745.0 cm^−1^ were assigned to MgF. In our spectra, three bands at 753.7, 750.3, and 746.5 cm^−1^ show an intensity ratio of about 8:1:1, indicative of a magnesium isotope pattern (natural abundances: 79% ^24^Mg, 10% ^25^Mg, 11% ^26^Mg).^[^ ^20^ ^]^ They are shifted to 739.5, 737.3, and 734.7 cm^−1^, respectively, in ^18^OF_2_ isotopic substitution experiments (Figure S2), evidencing that the responsible species contains oxygen and thus cannot be MgF. After irradiation with 273 nm the intensities of those peaks increased, while those of OF_2_ decreased (Figures S2 and S3). In the light of these new experimental results and supported by our calculations, we reassign these bands to side‐on F′Mg(η^2^‐OF). According to the calculation, this compound should have another intense transition at about 800 cm^−1^. However, due to overlap with the intense antisymmetric F─O─F stretching band of ^16/18^OF_2_ at 826/798 cm^−1^, it can be assigned only tentatively at 816.0/805.2 cm^−1^ for F′^24^Mg(η^2^–^16/18^OF) (Figure S3).

For calcium, besides the calcium fluorides, one new weak band at 784.1 cm^−1^ and a strong band at 548.0 cm^−1^ have been observed after the reaction of OF_2_ with Ca atoms. Upon ^16/18^O isotopic substitution, these bands shifted to 761.2 and 547.2 cm^−1^, respectively. Based on their positions and the corresponding isotopic shifts, they are assigned to the O─F and Ca─F′ stretching vibrations of the side‐on F′Ca(η^2^‐OF) species, respectively. They increased in intensity upon irradiation with 455 nm and full‐arc mercury lamp photolysis (*λ* > 220 nm) (Figure S4).

Similar to the calcium experiment, an intense SrF_2_ band at 448.2 cm^−1^ appeared after deposition.^[^ ^21^ ^]^ A new intense band at 454.1 cm^−1^ was observed in the Sr─F vibration region. A weak band at 786.9 cm^−1^ was also observed, which shifted to 763.4 cm^−1^ in the experiment with ^18^OF_2_. This set of absorptions increased after irradiation with 455 nm and was assigned to F′Sr(η^2^‐OF) (Figure S5). Also, a small amount of the free trifluoride anion [F_3_]^−^ occurred after irradiation with 455 nm light.^[^ ^22^ ^]^

In the experiment of laser‐ablated Ba atoms and OF_2_, a characteristic ^16/18^O isotope shift from 794.2 to 770.7 cm^−1^ could be observed (Figure S6). These bands have been assigned to the O─F stretching vibration of side‐on F′Ba(η^2^‐OF). Based on the calculations, the Ba─F′ vibration of side‐on F′Ba(η^2^‐OF) is expected to be below the detection range of the MCT detector (∼450 cm^−1^). To further confirm the formation of F′Ba(η^2^‐OF), far‐IR spectra were recorded using a bolometer detector cooled with liquid helium (Figure S7). The bands of BaF_2_ occur at 418.8 and 395.9 cm^−1^.^[^ ^21^ ^]^ In addition, a new band appeared at 351.8 cm^−1^ with a corresponding ^18^O isotopic band at 334.1 cm^−1^, which has been assigned to the Ba─O vibration of F′Ba(η^2^‐OF). The Ba─F′ absorption of F′Ba(η^2^‐OF) was at first challenging to assign since it overlapped with the symmetric stretching band of BaF_2_. However, it was later identified at 418.2 cm^−1^ from the difference spectra upon irradiation and annealing, as it exhibited different chemical behaviors from the BaF_2_ bands. The intensity of the F′Ba(η^2^‐OF) bands increased significantly after irradiation with 656 nm light and annealing to 15 K. In contrast, the BaF_2_ bands increased only slightly after irradiation with 656 nm and decreased after annealing to 15 K (Figure S7, b, and c). No detectable ^16/18^O isotopic shift was observed for the Ba─F′ band of F′Ba(η^2^‐OF). In the bolometer experiment with ^18^OF_2_, a band of SiF_4_ at 384.8 cm^−1^ also appeared.^[^ ^23^ ^]^ The SiF_4_ impurity arises due to the ^18^OF_2_ being synthesized and stored in glassware; over time, the ^18^OF_2_ reacted slowly with SiO_2_ to form SiF_4_. Unlike for many transition metals and beryllium,^[^ ^4^, ^5^, ^6^, ^7^, ^8^, ^9^, ^10^ ^]^ OMF was not observed for the heavier alkaline earth metals in any of our experiments (calculated data shown in Table S30).

The experimental and the calculated vibrational wavenumbers of side‐on F′M(η^2^‐OF) at B3LYP‐D3 and CCSD(T) levels of theory are summarized in Table 2. The experimental values agree with the calculated wavenumbers of side‐on hypofluorites F′M(η^2^‐OF) very well, which further supports our assignment. The increasing O─F stretching wavenumbers from Ca to Ba as well as ^16/18^O shifts for the Ca, Sr, and Ba species are similarly well reproduced at the B3LYP and CCSD(T) levels, while ^16/18^O shifts and ^24/25/26^Mg shifts for the Mg species are captured more accurately at the B3LYP level. The latter indicates that the extent of the strong coupling between the Mg─F′ and O─F modes is better captured at the B3LYP level, likely due to fortunate error cancelation between the electronic structure method and the harmonic approximation.

**Table 2:** Experimental and calculated vibrational wavenumbers ν in cm^−1^, IR intensities in km mol^−1^ (in parentheses), and ^16/18^O isotopic shifts Δν in cm^−1^ for side‐on F′M(η^2^‐OF) (M = Mg, Ca, Sr, Ba) species. ip: in‐phase coupling oop: out‐of‐phase coupling.

	Ar Matrix		B3LYP‐D3^a)^		CCSD(T)^b)^		
Species	ν(^16^O)	∆ν(^16/18^O)	ν(^16^O)	∆ν(^16/18^O)	ν(^16^O)	∆ν(^16/18^O)	Assignment
F′^24^Mg(η^2^‐OF)	753.7	−14.2	781.5 (80)	−15.3	768.1	−17.9	^24^Mg─F′/O─F ip
	816.0^c)^	−10.8^c)^	844.7 (102)	−10.9	845.8	−8.1	^24^Mg─F′/O─ F oop
F′^25^Mg(η^2^‐OF)	750.3	−13.0	777.9 (87)	−13.9	766.2	−17.2	^25^Mg─F′/O─F ip
	obscured	–	838.2 (91)	−12.3	837.4	−8.9	^25^Mg─F′/O─F oop
F′^26^Mg(η^2^‐OF)	746.5	−11.8	773.9 (93)	−12.5	764.1	−16.2	^26^Mg─F′/O─F ip
	obscured	–	832.8 (80)	−13.8	830.1	−9.8	^26^Mg─F′/O─F oop
F′Ca(η^2^‐OF)	548.0	−0.8	580.3 (259)	−1.2	584.0	−1.3	Ca─F′ str.
	784.1	−22.9	808.1 (35)	−23.7	785.8	−23.2	O─F str.
F′Sr(η^2^‐OF)	454.1	0.0	476.4 (147)	0.0	477.4	−0.1	Sr─F′ str.
	786.9	−23.5	811.8 (30)	−23.9	790.1	−23.5	O─F str.
F′Ba(η^2^‐OF)	351.8	−17.7	362.2 (88)	−18.2	364.6	−18.2	Ba─O str.
	418.2	0.0	436.7 (132)	−0.1	433.2	−0.2	Ba─F′ str.
	794.2	−23.5	826.0 (28)	−24.4	800.2	−23.8	O─F str.

^a)^ B3LYP‐D3/def2‐TZVP.

^b)^ CCSD(T)/awCVTZ‐(PP) without frozen core approximation of metals.

^c)^ Tentatively assigned.

The NBO analysis of side‐on F′M(η^2^‐OF) was carried out at B3LYP‐D3 level (Table S31). The NPA charge shows that the positive charge (≈ +1.8 *e*) is localized on the metal center, while the F′ and OF ligands carry approximately one negative elementary charge each. Moreover, the Wiberg bond orders show that only the O─F bond has a covalent character, with a bond order of approximately 0.9, indicating that the interactions between the metal atom and the ligands within the F′M(η^2^‐OF) molecule are mainly electrostatic. This is also supported by the AIM analysis (Table S32), which yields for all bond critical points between the metal and any other atom low electron densities but high, positive Laplacians, ELF values close to zero and ratios of the potential and kinetic energy densities close to or below 1.0—all characteristics of ionic bonds.

However, the electrostatic interaction cannot explain the non‐planar structure and different dihedral angles for different metal compounds. The extended transition state‐natural orbitals for chemical valence (ETS‐NOCV) analysis gives more detail on the nature of the side‐on coordination. F′M^+^ and OF^−^ are chosen as interacting fragments. The results in Table 3 show that the attraction between F′M^+^ and OF^−^ is primarily due to the Coulomb interaction, while less than 20% results from orbital interactions, consistent with the NBO results.

**Table 3:** Results of the ETS‐NOCV analysis for F′M(η^2^‐OF) (M = Mg, Ca, Sr, Ba) using F′M^+^ and OF^−^ as interacting fragments at the B3LYP‐D3(BJ)/TZ2P//B3LYP‐D3/def2‐TZVP level. Interaction energies in kJ mol^−1^.

	F′Mg(η^2^‐OF)	F′Ca(η^2^‐OF)	F′Sr(η^2^‐OF)	F′Ba(η^2^‐OF)
Δ*E*_tot_	−959.3	−792.1	−735.2	−689.5
Δ*E*_pauli_	221.7	270.3	262.4	283.5
Δ*E*_elstat_	−990.6 (84%)	−879.1 (83%)	−832.3 (84%)	−790.2 (82%)
Δ*E*_orb_	−186.0 (16%)	−179.2 (17%)	−161.2 (16%)	−175.5 (18%)
Δ*E*_orb(1)_	−69.8	−75.2	−70.2	−84.3
Δ*E*_orb(2)_	−38.0	−31.5	−27.4	−28.8
Δ*E*_orb(3)_	−29.2	−37.6	−33.1	−32.9
Δ*E*_orb(rest)_	−50.0	−34.9	−30.5	−29.5

**Figure 4:**
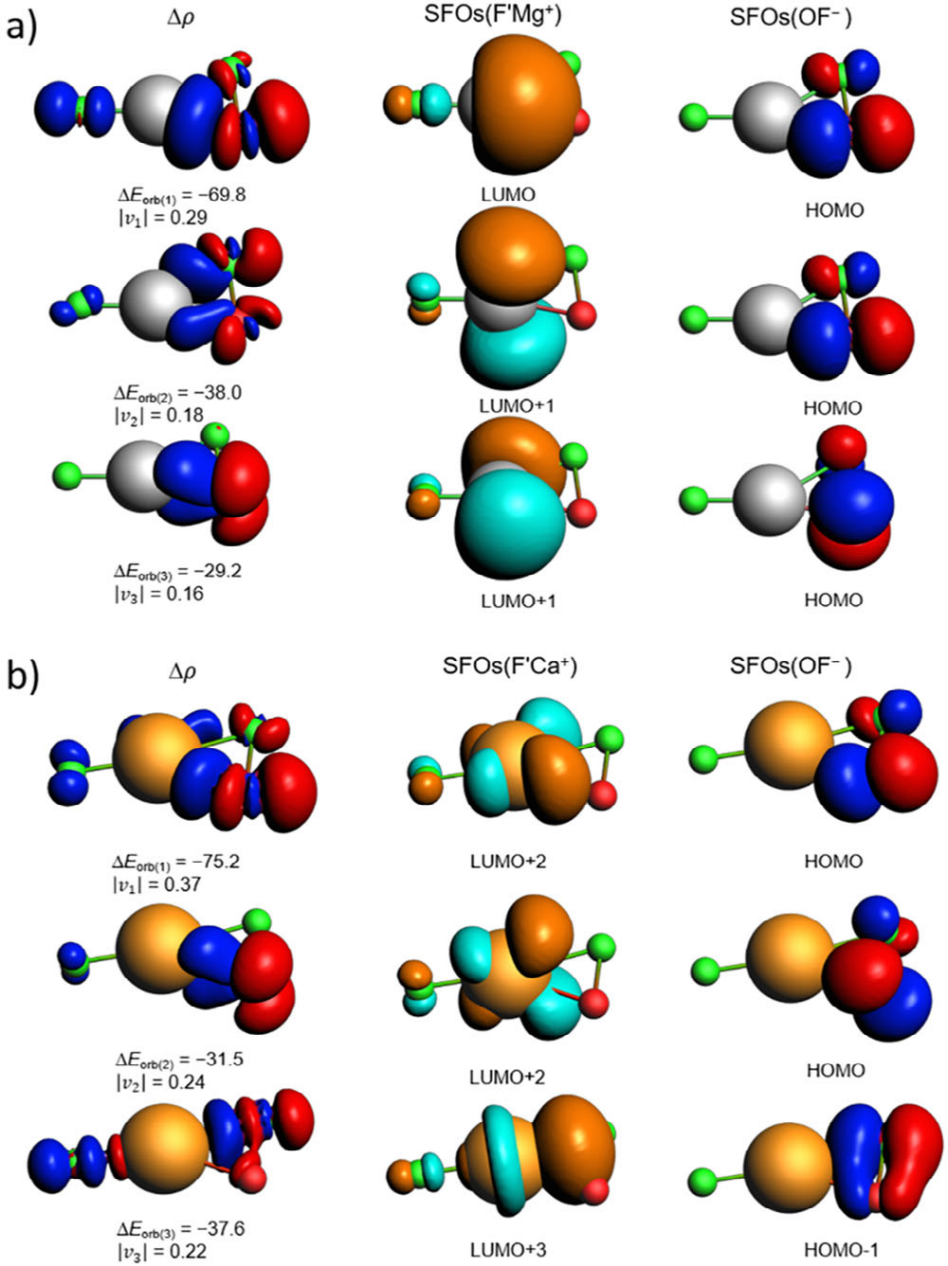
Deformation densities ∆*ρ* (left column), corresponding eigenvalues ν and Δ*E*_orb_ interaction energies in kJ mol^−1^, as well as shape of the most relevant respective symmetrized fragment orbitals (SFOs) of the fragments F′M^+^ (middle column) and OF^−^ (right column) from ETS‐NOCV calculations for a) F′Mg(η^2^‐OF) and b) F′Ca(η^2^‐OF) performed at B3LYP‐D3(BJ)/TZ2P//B3LYP‐D3/def2‐TZVP level. The isosurface values for deformation densities ∆*ρ* is 0.001 au, and the electronic charge flow is red to blue. The isosurface values for the SFOs is 0.05 au.

In terms of orbital interaction, there are three relatively strong pairwise orbital interactions Δ*E*_orb(1)_−Δ*E*_orb(3)_. The corresponding deformation densities are shown in Figure 4 for F′Mg(η^2^‐OF) and F′Ca(η^2^‐OF), and in Figures S8 and S9 for F′Sr(η^2^‐OF) and F′Ba(η^2^‐OF). The orbital interactions within the F′Mg(η^2^‐OF) species are mainly due to the donation of the π* orbital of OF^−^ to the empty s and p orbitals of the Mg center, while for F′Ca(η^2^‐OF), F′Sr(η^2^‐OF), and F′Ba(η^2^‐OF) the orbital interactions are based on the donation of occupied orbitals of OF^−^ to the vacant d orbitals of the metal center. For magnesium, the structure agrees well with the VSEPR‐model, as its valence s and p orbitals favor the formation of a linear (planar) structure.

In contrast, for the heavy alkaline earth metals Ca, Sr, and Ba, the participation of d orbitals in the orbital interactions results in a shallow bending potential or a non‐planar geometry, since a d orbital prefers to participate in a bent (non‐planar) structure. Moreover, the increasing polarizability of the metal cation from Ca to Ba promotes a more pyramidal structure. This type of non‐VSEPR structure is common for complexes where the metal center has a formal d^0^ electronic configuration.^[^ ^24^ ^]^

Some parallels can be drawn to the molecular alkaline earth metal dicyanides, for which the heavier members (Ca–Ba) also exhibit side‐on coordination and they all display a non‐planar structure.^[^ ^25^ ^]^ However, magnesium can only form linear di‐isocyanide, unlike the hypofluorite. Overall, this appears less surprising considering that CN^−^ is well‐known as an ambidentate ligand, unlike OF^−^.

Mercury, which has some similar characteristics to alkaline earth metals,^[^ ^2^ ^]^ can also form the insertion hypofluorite product FHg(η^1^‐OF), with Hg in a +II oxidation state, but does so with an end‐on structure and a HgOF angle of 102° at CCSD(T)/aug‐cc‐pVTZ level,^[^ ^1^ ^]^ perhaps the consequence of a more covalent Hg─O bond and the fully occupied d orbitals of mercury. Molecular oxyfluorides of the lighter group 12 metals Zn and Cd appear to be unexplored so far.

## Conclusion

In conclusion, laser‐ablated alkaline earth metal atoms Mg, Ca, Sr, and Ba react with OF_2_ to form side‐on hypofluorites F′M(η^2^‐OF) in rare gas matrices, in contrast to all previously reported metal oxyfluorides formed by the reaction of metal with OF_2_. This includes the reassignment of bands previously attributed to MgF. The M─F─O─F′ dihedral angle increases from Ca to Ba, which parallels the angle changing of alkaline earth metal dihydrides and difluorides MX_2_ (X = H, F), where BeX_2_ and MgX_2_ are linear, while CaX_2_ is quasi‐linear and SrX_2_ and BaX_2_ are bent. It shows that non‐VSEPR structures are realized not only in simple homoleptic alkaline earth metal compounds, but also in heteroleptic and polyatomic ones. Quantum‐chemical calculations reveal that the bonding interactions are mainly due to the electrostatic interactions between the metal center M^2+^ and the negatively charged ligands F′^−^ and OF^−^. For F′Mg(η^2^‐OF), the orbital interactions come from the donation of electron density from the π* orbital of the OF^−^ to the empty s and p orbitals of magnesium, while for F′Ca(η^2^‐OF), F′Sr(η^2^‐OF), and F′Ba(η^2^‐OF), the orbital interactions are due to the donation from occupied orbitals of OF^−^ to the vacant d orbitals of the metal center. The participation of d orbitals in the orbital interaction contributes to a shallow bending potential or a non‐planar structure of the heavy alkaline earth metal compounds. This further highlights the significance of the d orbitals in heavy alkaline earth metal chemistry.

## Supporting Information

The authors have cited additional references within the Supporting Information.^[^ ^18^, ^26^, ^27^, ^28^, ^29^, ^30^, ^31^, ^32^, ^33^, ^34^, ^35^, ^36^, ^37^, ^38^, ^39^, ^40^, ^41^, ^42^, ^43^, ^44^, ^45^, ^46^ ^]^

## Conflict of Interests

The authors declare no conflict of interest.

## Supporting information



Supporting Information

## Data Availability

The data that support the findings of this study are available from the corresponding author upon reasonable request.
