# Supplementary material for: Side‐On Coordination in Molecular Alkaline Earth Metal Hypofluorites FM(η2‐OF) (M = Mg, Ca, Sr, and Ba)
Source: Angew Chem Int Ed Engl. 2025 Jul 16;64(35):e202507151. doi: 10.1002/anie.202507151 (PMC12377426; doi:10.1002/anie.202507151)
Supplement: Supplementary file 1 — Supporting Information [file ANIE-64-e202507151-s001.docx]

Supporting Information
©Wiley-VCH 2021
69451 Weinheim, Germany

Side-on Coordination in Molecular Alkaline Earth Metal Hypofluorites FM(η^2^-OF) (M = Mg, Ca, Sr and Ba)

Xiya Xia, Robert Medel, Sebastian Riedel*

**Abstract:** Side-on coordinated molecular hypofluorites FM(η^2^-OF) (M = Mg, Ca, Sr, and Ba) were prepared by co-deposition of laser-ablated alkaline earth metal atoms with diluted OF_2_ in cryogenic matrices. The structures parallel the non-VESPR structures of alkaline earth metal difluorides and dihydrides, such that FMg(η^2^-OF) is planar, FCa(η^2^-OF) is planar but with a shallow bending-potential curve, while FSr(η^2^-OF) and FBa(η^2^-OF) are non-planar. Bonding analyses show that the interactions between the metal center M^2+^ and the negatively charged ligands F^−^ and OF^−^ are mainly electrostatic. Moreover, for FMg(η^2^-OF), donation of electron density from OF^−^ to empty s and p orbitals of magnesium is involved. However, for the heavier alkaline earth metal compounds, the donation to empty d orbitals of the metal center becomes dominant in orbital interactions, which contributes to their non-planar structures.

Table of Contents

[Experimental Procedures 3](#_Toc191908821)

[Computational Procedures 3](#_Toc191908822)

[Results and Discussion 4](#_Toc191908823)

[Calculated reaction energies 4](#_Toc191908824)

[Calculated relative isomer energy 5](#_Toc191908825)

[Calculated vibrational data 6](#_Toc191908826)

[IR spectra 15](#_Toc191908827)

[Bonding analysis data 20](#_Toc191908828)

[ETS-NOCV analysis 21](#_Toc191908829)

[Calculated molecular structures 22](#_Toc191908830)

[References 30](#_Toc191908831)

[Author Contributions 30](#_Toc191908832)

Experimental Procedures

The alkali earth metal oxyfluoride molecules were synthesized by co-depositing laser-ablated Mg, Ca, Sr or Ba atoms with ^16/18^OF_2_ diluted in argon (99.999 %, Sauerstoffwerk Friedrichshafen). The gas mixtures were prepared in a stainless-steel mixing chamber equipped with a manometer, and then transferred to the custom-built matrix chamber through a stainless-steel capillary. The deposition occurred on a gold-plated copper mirror cooled by a closed-cycle helium cryostat (Sumitomo Heavy Industries, RDK-205D) to 10 K inside the matrix chamber.

^16/18^OF_2_ was synthesized by the reaction of elemental fluorine with ^16/18^OH_2_ dispersed in solid KF. FTIR spectra were recorded on a Bruker Vertex 80v spectrometer at 0.5 cm^−1^ resolution with 200 scans. A liquid-nitrogen-cooled mercury cadmium telluride (MCT) detector was used for recording mid-infrared MIR spectra in the 4000−450 cm^−1^ region, while a liquid-helium-cooled bolometer was employed for far-infrared FIR spectra in the 700–180 cm^−1^ region. The FTIR spectra after co-deposition of laser-ablated alkaline earth metals with diluted F_2_ were recorded on a similar apparatus but with a Bruker Vertex 70 spectrometer and a CsI window in transmission mode.

Laser ablation was performed using a Continuum Inc. Minilite II Nd laser (*λ* = 1064 nm), operating at a repetition rate of 10 Hz, a pulse length of 10 ns, and a variable pulse energy from 50−60 mJ. The laser beam was focused onto the target using a Thorlabs N-BK7 (LA1986-C) plano-convex lens with a focal distance of 125.0 mm.

The matrix samples were annealed at different temperatures and irradiated by LED lights with different wavelength (656 nm, 455 nm, 273 nm) as well as by a full-arc mercury lamp with the outer globe removed (*λ* > 220 nm).

Computational Procedures

Density functional theory (DFT) calculations for optimization of the structures and harmonic vibrational frequencies were performed using the Gaussian16, Revision A.03 program package.^[26]^ The hybrid functional B3LYP^[27–30]^ with the original Grimme’s empirical dispersion D3 correction^[31]^ and def2-TZVP^[18,32]^ basis set were employed. The Natural Bond Orbital analysis (NBO) analyses were done by NBO 7.0.^[33]^ QTAIM analyses were carried out by Multiwfn Version 3.8.^[34]^ The extended transition state-natural orbitals for chemical valence (ETS-NOCV) analyses^[35–37]^ were performed in ADF software in ams2024.106.^[38]^

CCSD(T) calculations were carried out using Molpro 2021.3.^[39–41]^ The augmented triple-ζ basis sets aug-cc-pVTZ were used for oxygen, fluorine,^[42,43]^ the aug-cc-pVTZ and the augmented correlation consistent polarized weighted core valence triple-ζ basis set aug-cc-pwCVTZ were used for magnesium,^[44]^ and the aug-cc-pVTZ-PP or aug-cc-pwCVTZ-PP valence basis with the corresponding scalar-relativistic pseudopotentials (PP) for calcium, strontium and barium with and without the frozen core approximation.^[45,46]^

Results and Discussion

Calculated reaction energies

**Scheme S1**. Reaction energy of formation of FM(η^2^-OF) from metal atoms and OF_2_, calculated at B3LYP-D3/def2-TZVP (all metals), as well as CCSD(T)/awCVTZ for Mg and CCSD(T)/awCVTZ-PP for Ca, Sr, Ba without frozen core of metals.

**Scheme S2**. Dissociation energy from FM(η^2^-OF) to OMF, calculated at B3LYP-D3/def2-TZVP (all metals), as well as CCSD(T)/awCVTZ for Mg and CCSD(T)/awCVTZ-PP for Ca, Sr, Ba without frozen core of metals.

Calculated relative isomer energy

Comparing side-on and end-on singlet structures of F'CaOF, F'SrOF and F'BaOF (Table S1), the B3LYP-D3 results indicate that all side-on structures are energetically lower. However, the CCSD(T) results demonstrate different trends depending on the treatment of the metal center. When the aug-cc-pVTZ-PP (aVTZ-PP) basis set is applied using the default frozen core approximation in Molpro 2021.3 (i.e. 3s3p for calcium, 4s4p for strontium and 5s5p for barium, lower orbitals are treated implicitly in the pseudo potential), the end-on products are calculated to be more stable and side-on F'Ca(η^2^-OF) even has one small imaginary frequency. When the frozen core approximation is turned off for the metal center while the aVTZ-PP basis is retained, all side-on products are found to have lower energy, indicating that the subvalence orbitals of heavy alkaline earth metals have a significant impact on computed energies. Therefore, the core-valence correlation might need to be further considered by using the aug-cc-pwCVTZ-PP (awCVTZ-PP) basis set. When combined the awCVTZ-PP basis set with the frozen core approximation, side-on F'Ca(η^2^-OF) becomes favorable but end-on F'SrOF and F'BaOF are still energetically lower, however the energy difference between two structures becomes smaller. With the combination of the awCVTZ-PP basis set and unfrozen core of the metals, all side-on structures become energetically favored and the energy differences between end-on and side-on structures are larger compared to the results obtained with the aVTZ-PP basis set. To determine which isomer is actually formed and which method is most suitable for this system, we next turn to experiment.

**Table S1.** Energy differences between end-on and side-on singlet isomers of F'MOF (M = Ca, Sr, Ba) in kJ mol^−1^ calculated at different levels of theory. Positive values indicate that the side-on structure is more stable. All energies are corrected by harmonic zero-point energy.

| Methods | F'CaOF | F'SrOF | F'BaOF |
| --- | --- | --- | --- |
| B3LYP-D3/  def2-TZVP | 38.3 | 32.6 | 24.2 |
| CCSD(T)/  aVTZ-PP^a^ | −24.3^c^ | −85.7 | −60.5 |
| CCSD(T)/  aVTZ-PP^b^ | 44.8 | 37.7 | 29.2 |
| CCSD(T)/  awCVTZ-PP^a^ | 12.6 | −13.5 | −26.3 |
| CCSD(T)/  awCVTZ-PP^b^ | 47.3 | 39.5 | 30.8 |

^a^ with frozen core of metals.  ^b^ without frozen core of metals.  ^c^ with imaginary frequency.

**Table S2.** Energy differences between singlet side-on isomers F'M(η^2^-OF) and triplet OMF_2_ (M = Mg, Ca, Sr, Ba) in kJ mol^−1^ calculated at CCSD(T)/awCVTZ(-PP) level, unless stated otherwise. Positive values indicate that the singlet side-on structure is more stable. All energies are corrected by harmonic zero-point energy.

| Metals | Energy difference |
| --- | --- |
| Mg | 2.8 |
| Ca | 22.1 |
| Sr | 23.3 |
| Ba | 24.9^a^ |

^a^ OBaF_2_ has one imaginary frequency.

Calculated vibrational data

**Table S3.** Calculated harmonic vibrational wavenumbers$\nu$ in cm^−1^, IR intensities in km mol^−1^ (in parentheses) and ^16/18^O isotopic shifts $\Delta\nu$ in cm^−1^ for side-on FMg(η^2^-OF) species at B3LYP-D3/def2-TZVP level.

| **side-on FMg(η^2^-OF) (^1^A')** | | | | | | |
| --- | --- | --- | --- | --- | --- | --- |
| **Symmetry** | **^24^Mg** | | **^25^Mg** | | **^26^Mg** | |
|  | $\nu$(^16^O) | ∆$\nu$(^16/18^O) | $\nu$(^16^O) | ∆$\nu$(^16/18^O) | $\nu$(^16^O) | ∆$\nu$(^16/18^O) |
| A' | 134.30 (79) | −0.67 | 133.05 (78) | −0.66 | 131.87 (77) | −0.65 |
| A'' | 149.78 (114) | −1.04 | 147.82 (111) | −1.06 | 145.99 (108) | −1.08 |
| A' | 320.91 (17) | −1.87 | 319.26 (16) | −1.85 | 317.74 (16) | −1.85 |
| A' | 505.44 (3) | −17.35 | 505.01 (3) | −17.45 | 504.61 (3) | −17.56 |
| A' | 781.52 (80) | −15.34 | 777.91 (87) | −13.94 | 773.86 (93) | −12.47 |
| A' | 844.71 (102) | −10.92 | 838.18 (91) | −12.32 | 832.83 (80) | −13.80 |

**Table S4.** Calculated harmonic vibrational wavenumbers $\nu$ in cm^−1^, IR intensities in km mol^−1^ (in parentheses) for end-on FMgOF species (not a true minimum with imaginary frequencies) at B3LYP-D3/def2-TZVP level.

| **end-on FMgOF** **(^1^∑^+^)** | | | |
| --- | --- | --- | --- |
| **Symmetry** | **^24^Mg** | **^25^Mg** | **^26^Mg** |
| Π | −162.84 (21) | −162.7 (21) | −162.57 (21) |
| Π | −162.84 (21) | −162.7 (21) | −162.57 (21) |
| Π | 126.41 (93) | 124.86 (91) | 123.42 (89) |
| Π | 126.41 (93) | 124.86 (91) | 123.42 (89) |
| Σ^+^ | 442.35 (10) | 441.62 (10) | 440.89 (11) |
| Σ^+^ | 797.96 (154) | 789.22 (150) | 781.04 (146) |
| Σ^+^ | 1215.91 (15) | 1214.23 (14) | 1212.73 (13) |

**Table S5.** Calculated harmonic vibrational wavenumbers$\nu$ in cm^−1^, IR intensities in km mol^−1^ (in parentheses) and ^16/18^O isotopic shifts $\Delta\nu$ in cm^−1^ for triplet OMgF_2_ species at B3LYP-D3/def2-TZVP level.

| **OMgF_2_ (^3^A'')** | | | | | | |
| --- | --- | --- | --- | --- | --- | --- |
| **Symmetry** | **^24^Mg** | | **^25^Mg** | | **^26^Mg** | |
|  | $\nu$(^16^O) | ∆$\nu$(^16/18^O) | $\nu$(^16^O) | ∆$\nu$(^16/18^O) | $\nu$(^16^O) | ∆$\nu$(^16/18^O) |
| A' | 122.39 (35) | −2.07 | 121.58 (35) | −2.04 | 120.80 (34) | −2.00 |
| A'' | 151.09 (116) | −0.20 | 149.05 (113) | −0.20 | 147.15 (110) | −0.19 |
| A' | 241.36 (65) | −5.11 | 240.91 (65) | −5.04 | 240.48 (64) | −4.98 |
| A' | 335.68 (3) | −11.92 | 333.65 (3) | −12.05 | 331.78 (3) | −12.19 |
| A' | 525.41 (5) | −0.42 | 524.86 (5) | −0.44 | 524.35 (5) | −0.45 |
| A' | 818.98 (144) | −0.33 | 809.03 (140) | −0.33 | 799.79 (137) | −0.33 |

**Table S6.** Calculated harmonic vibrational wavenumbers$\nu$ in cm^−1^, IR intensities in km mol^−1^ (in parentheses) and ^16/18^O isotopic shifts $\Delta\nu$ in cm^−1^ for side-on FCa(η^2^-OF) (^1^A') species at B3LYP-D3/def2-TZVP level.

| Symmetry | $\nu$(^16^O) | ∆$\nu$(^16/18^O) |
| --- | --- | --- |
| A" | 12.00 (82) | 0 |
| A' | 64.32 (69) | −0.48 |
| A' | 299.57 (8) | −1.21 |
| A' | 446.98 (28) | −17.60 |
| A' | 580.33 (259) | −1.18 |
| A' | 808.05 (35) | −23.67 |

**Table S7.** Calculated harmonic vibrational wavenumbers$\nu$ in cm^−1^, IR intensities in km mol^−1^ (in parentheses) and ^16/18^O isotopic shifts $\Delta\nu$ in cm^−1^ for end-on FCaOF (^1^∑^+^) species at B3LYP-D3/def2-TZVP level.

| Symmetry | $\nu$(^16^O) | ∆$\nu$(^16/18^O) |
| --- | --- | --- |
| Π | 14.63 (52) ×2 | 0 |
| Π | 170.92 (52) ×2 | −6.89 |
| Σ^+^ | 378.57 (25) | −4.17 |
| Σ^+^ | 577.34 (290) | −0.02 |
| Σ^+^ | 1077.90 (0.1) | −39.56 |

**Table S8.** Calculated harmonic vibrational wavenumbers$\nu$ in cm^−1^, IR intensities in km mol^−1^ (in parentheses) and ^16/18^O isotopic shifts $\Delta\nu$ in cm^−1^ for triplet OCaF_2_ (^3^A) species at B3LYP-D3/def2-TZVP level.

| Symmetry | $\nu$(^16^O) | ∆$\nu$(^16/18^O) |
| --- | --- | --- |
| A | 40.65 (76) | −0.41 |
| A | 70.79 (59) | −0.49 |
| A | 247.62 (42) | −5.40 |
| A | 267.57 (2) | −11.46 |
| A | 456.50 (29) | −1.06 |
| A | 578.67 (253) | −0.24 |

**Table S9.** Calculated harmonic vibrational wavenumbers$\nu$ in cm^−1^, IR intensities in km mol^−1^ (in parentheses) and ^16/18^O isotopic shifts $\Delta\nu$ in cm^−1^ for side-on FSr(η^2^-OF) (^1^A) species at B3LYP-D3/def2-TZVP level.

| Symmetry | $\nu$(^16^O) | ∆$\nu$(^16/18^O) |
| --- | --- | --- |
| A | 38.83 (29) | −0.12 |
| A | 70.32 (42) | −1.17 |
| A | 264.42 (15) | −0.9 |
| A | 396.80 (59) | −18.81 |
| A | 476.42 (147) | −0.02 |
| A | 811.76 (30) | −23.93 |

**Table S10.** Calculated harmonic vibrational wavenumbers$\nu$ in cm^−1^, IR intensities in km mol^−1^ (in parentheses) and ^16/18^O isotopic shifts $\Delta\nu$ in cm^−1^ for end-on FSrOF (^1^A') species at B3LYP-D3/def2-TZVP level.

| Symmetry | $\nu$(^16^O) | ∆$\nu$(^16/18^O) |
| --- | --- | --- |
| A' | 43.08 (27) | −0.02 |
| A'' | 151.08 (34) | −6.46 |
| A' | 154.17 (32) | −6.41 |
| A' | 337.06 (68) | −5.62 |
| A' | 475.95 (164) | −0.01 |
| A' | 1037.66 (5) | −37.04 |

**Table S11.** Calculated harmonic vibrational wavenumbers$\nu$ in cm^−1^, IR intensities in km mol^−1^ (in parentheses) and ^16/18^O isotopic shifts $\Delta\nu$ in cm^−1^ for triplet OSrF_2_ (^3^A) species at B3LYP-D3/def2-TZVP level.

| Symmetry | $\nu$(^16^O) | ∆$\nu$(^16/18^O) |
| --- | --- | --- |
| A | 46.00 (33) | −0.94 |
| A | 73.43 (41) | −0.08 |
| A | 221.55 (9) | −8.97 |
| A | 257.97 (38) | −7.49 |
| A | 409.16 (61) | −1.89 |
| A | 475.48 (143) | −0.01 |

**Table S12.** Calculated harmonic vibrational wavenumbers$\nu$ in cm^−1^, IR intensities in km mol^−1^ (in parentheses) and ^16/18^O isotopic shifts $\Delta\nu$ in cm^−1^ for side-on FBa(η^2^-OF) (^1^A) species at B3LYP-D3/def2-TZVP level.

| Symmetry | $\nu$(^16^O) | ∆$\nu$(^16/18^O) |
| --- | --- | --- |
| A | 37.36 (13) | −0.24 |
| A | 76.39 (26) | −1.49 |
| A | 232.52 (15) | −0.74 |
| A | 362.00 (88) | −18.15 |
| A | 436.74 (132) | −0.14 |
| A | 826.01 (28) | −24.43 |

**Table S13.** Calculated harmonic vibrational wavenumbers$\nu$ in cm^−1^, IR intensities in km mol^−1^ (in parentheses) and ^16/18^O isotopic shifts $\Delta\nu$in cm^−1^ for end-on FBaOF (^1^A') species at B3LYP-D3/def2-TZVP level.

| Symmetry | $\nu$(^16^O) | ∆$\nu$(^16/18^O) |
| --- | --- | --- |
| A' | 50.18 (17) | −0.03 |
| A'' | 129.58 (24) | −5.20 |
| A' | 134.51 (28) | −5.73 |
| A' | 306.08 (89) | −6.68 |
| A' | 435.48 (146) | −0.04 |
| A' | 999.62 (10) | −34.58 |

**Table S14.** Calculated harmonic vibrational wavenumbers$\nu$ in cm^−1^, IR intensities in km mol^−1^ (in parentheses) and ^16/18^O isotopic shifts $\Delta\nu$ in cm^−1^ for triplet OBaF_2_ (^3^A) species at B3LYP-D3/def2-TZVP level.

| Symmetry | $\nu$(^16^O) | ∆$\nu$(^16/18^O) |
| --- | --- | --- |
| A | 43.84 (17) | −1.09 |
| A | 79.85 (27) | −0.02 |
| A | 187.01 (3) | −8.64 |
| A | 253.51 (54) | −6.00 |
| A | 375.85 (86) | −2.46 |
| A | 436.51 (126) | −0.01 |

| **side-on FMg(η^2^-OF) (^1^A')** | | | | | | |
| --- | --- | --- | --- | --- | --- | --- |
| **symmetry** | **^24^Mg** | | **^25^Mg** | | **^26^Mg** | |
|  | $\nu$(^16^O) | ∆$\nu$(^16/18^O) | $\nu$(^16^O) | ∆$\nu$(^16/18^O) | $\nu$(^16^O) | ∆$\nu$(^16/18^O) |
| A' | 132.11 | −0.68 | 130.88 | −0.66 | 129.73 | −0.65 |
| A'' | 142.06 | −0.96 | 140.19 | −0.97 | 138.46 | −0.99 |
| A' | 334.33 | −1.77 | 332.62 | −1.77 | 331.02 | −1.77 |
| A' | 500.24 | −17.22 | 499.77 | −17.32 | 499.32 | −17.42 |
| A' | 764.23 | −16.98 | 761.77 | −15.93 | 758.95 | −14.76 |
| A' | 832.95 | −8.82 | 825.4 | −9.86 | 818.93 | −11.04 |

**Table S15.** Calculated harmonic vibrational wavenumbers$\nu$ in cm^−1^ and ^16/18^O isotopic shifts $\Delta\nu$in cm^−1^ for side-on FMg(η^2^-OF) species at CCSD(T)/aVTZ level with frozen core of Mg.

**Table S16.** Calculated harmonic vibrational wavenumbers$\nu$ in cm^−1^ and ^16/18^O isotopic shifts $\Delta\nu$in cm^−1^ for end-on FMgOF species at CCSD(T)/aVTZ level with frozen core of Mg.

| **end-on FMgOF (^1^∑^+^)** | | |
| --- | --- | --- |
| **^24^Mg** | **^25^Mg** | **^26^Mg** |
| −153.21 | −153.09 | −152.98 |
| −153.21 | −153.09 | −152.98 |
| 122.76 | 121.25 | 119.85 |
| 122.76 | 121.25 | 119.85 |
| 434.66 | 433.89 | 433.13 |
| 788.78 | 780.45 | 772.65 |
| 1173.46 | 1171.5 | 1169.76 |

**Table S17.** Calculated harmonic vibrational wavenumbers$\nu$ in cm^−1^ and ^16/18^O isotopic shifts $\Delta\nu$ in cm^−1^ for triplet OMgF_2_ species at CCSD(T)/aVTZ level with frozen core of Mg.

| **OMgF_2_ (^3^A'')** | | | | | | |
| --- | --- | --- | --- | --- | --- | --- |
| **Symmetry** | **^24^Mg** | | **^25^Mg** | | **^26^Mg** | |
|  | $\nu$(^16^O) | ∆$\nu$(^16/18^O) | $\nu$(^16^O) | ∆$\nu$(^16/18^O) | $\nu$(^16^O) | ∆$\nu$(^16/18^O) |
| A' | 39.17 | −1.09 | 39.11 | −1.08 | 39.06 | −1.09 |
| A'' | 144.98 | −3.44 | 144.51 | −2.89 | 144.05 | −2.80 |
| A' | 154.26 | −0.79 | 152.25 | −0.06 | 150.37 | −0.06 |
| A' | 261.45 | −7.33 | 258.76 | −6.72 | 256.27 | −6.84 |
| A' | 551.42 | −0.18 | 551.15 | 0.00 | 550.91 | 0.00 |
| A' | 844.54 | −0.11 | 834.21 | 0.00 | 824.61 | −0.01 |

| **side-on FMg(η^2^-OF) (^1^A')** | | | | | | |
| --- | --- | --- | --- | --- | --- | --- |
| **symmetry** | **^24^Mg** | | **^25^Mg** | | **^26^Mg** | |
|  | $\nu$(^16^O) | ∆$\nu$(^16/18^O) | $\nu$(^16^O) | ∆$\nu$(^16/18^O) | $\nu$(^16^O) | ∆$\nu$(^16/18^O) |
| A' | 134.54 | −0.70 | 133.30 | −0.69 | 132.13 | −0.68 |
| A'' | 145.44 | −0.97 | 143.52 | −0.98 | 141.74 | −0.99 |
| A' | 341.93 | −1.76 | 340.18 | −1.76 | 338.55 | −1.76 |
| A' | 513.15 | −17.58 | 512.64 | −17.68 | 512.16 | −17.78 |
| A' | 768.13 | −17.94 | 766.25 | −17.15 | 764.07 | −16.22 |
| A' | 845.72 | −8.10 | 837.42 | −8.91 | 830.12 | −9.82 |

**Table S18.** Calculated harmonic vibrational wavenumbers$\nu$ in cm^−1^ and ^16/18^O isotopic shifts $\Delta\nu$in cm^−1^ for side-on FMg(η^2^-OF) species at CCSD(T)/awCVTZ level without frozen core of Mg.

**Table S19.** Calculated harmonic vibrational wavenumbers$\nu$ in cm^−1^ and ^16/18^O isotopic shifts $\Delta\nu$in cm^−1^ for end-on FMgOF species at CCSD(T)/awCVTZ level without frozen core of Mg.

| **end-on FMgOF (^1^∑^+^)** | | |
| --- | --- | --- |
| **^24^Mg** | **^25^Mg** | **^26^Mg** |
| −137.99 | −137.93 | −137.86 |
| −137.99 | −137.93 | −137.86 |
| 128.05 | 126.44 | 124.95 |
| 128.05 | 126.44 | 124.95 |
| 445.17 | 444.40 | 443.64 |
| 802.87 | 794.48 | 786.61 |
| 1186.11 | 1183.95 | 1182.02 |

**Table S20.** Calculated harmonic vibrational wavenumbers$\nu$ in cm^−1^ and ^16/18^O isotopic shifts $\Delta\nu$ in cm^−1^ for triplet OMgF_2_ species at CCSD(T)/awCVTZ level without frozen core of Mg.

| **OMgF_2_ (^3^A'')** | | | | | | |
| --- | --- | --- | --- | --- | --- | --- |
| **Symmetry** | **^24^Mg** | | **^25^Mg** | | **^26^Mg** | |
|  | $\nu$(^16^O) | ∆$\nu$(^16/18^O) | $\nu$(^16^O) | ∆$\nu$(^16/18^O) | $\nu$(^16^O) | ∆$\nu$(^16/18^O) |
| A' | 40.43 | −1.10 | 40.36 | −1.10 | 40.29 | −1.10 |
| A'' | 148.44 | −2.98 | 147.93 | −2.88 | 147.44 | −2.79 |
| A' | 153.94 | −0.06 | 151.92 | −0.06 | 150.05 | −0.06 |
| A' | 276.51 | −7.15 | 273.72 | −7.29 | 271.14 | −7.42 |
| A' | 560.22 | 0.00 | 559.93 | 0.00 | 559.67 | 0.00 |
| A' | 856.45 | 0.00 | 845.98 | 0.00 | 836.25 | 0.00 |

**Table S21.** Calculated harmonic vibrational wavenumbers$\nu$ in cm^−1^ and ^16/18^O isotopic shifts $\Delta\nu$in cm^−1^ for side-on FCa(η^2^-OF) species at CCSD(T) level.

| **side-on FCa(η^2^-OF) (^1^A')** | | | | | | | | |
| --- | --- | --- | --- | --- | --- | --- | --- | --- |
| Symmetry | aVTZ-PP^b^ | | aVTZ-PP^c^ | | awCVTZ-PP^b^ | | awCVTZ-PP^c^ | |
|  | $\nu$(^16^O) | $\nu$(^16^O) | $\nu$(^16^O) | ∆$\nu$(^16/18^O) | $\nu$(^16^O) | ∆$\nu$(^16/18^O) | $\nu$(^16^O) | ∆$\nu$(^16/18^O) |
| A" | −53.94^a^ | 0.68 | 36.87 | −0.13 | 19.00 | 0 | 42.13 | −0.13 |
| A' | 63.08 | −0.36 | 69.30 | −0.45 | 62.86 | −0.42 | 63.91 | −0.43 |
| A' | 320.12 | −0.91 | 324.09 | −0.98 | 320.87 | −1.05 | 320.43 | −1.04 |
| A' | 428.72 | −17.26 | 436.92 | −17.6 | 437.99 | −17.55 | 442.7 | −17.64 |
| A' | 583.65 | −0.65 | 580.53 | −1.26 | 589.77 | −1.07 | 584.04 | −1.32 |
| A' | 788.92 | −23.33 | 784.63 | −23.23 | 791.82 | −23.44 | 785.79 | −23.25 |

^a^ with imaginary frequency.  ^b^ with frozen core of metals.  ^c^ without frozen core of metals.

**Table S22.** Calculated harmonic vibrational wavenumbers$\nu$ in cm^−1^ and ^16/18^O isotopic shifts $\Delta\nu$ in cm^−1^ for end-on FCaOF species at CCSD(T) level.

| **end-on FCaOF (^1^∑^+^)** | | | | | | | | |
| --- | --- | --- | --- | --- | --- | --- | --- | --- |
| Symmetry | aVTZ-PP^a^ | | aVTZ-PP^b^ | | awCVTZ-PP^a^ | | awCVTZ-PP^b^ | |
|  | $\nu$(^16^O) | ∆$\nu$(^16/18^O) | $\nu$(^16^O) | ∆$\nu$(^16/18^O) | $\nu$(^16^O) | ∆$\nu$(^16/18^O) | $\nu$(^16^O) | ∆$\nu$(^16/18^O) |
| Π | 69.76×2 | −0.35 | 62.20×2 | −0.14 | 66.14×2 | −0.31 | 39.63×2 | −0.05 |
| Π | 177.09×2 | −6.85 | 221.77×2 | −8.78 | 163.07×2 | −6.32 | 172.72×2 | −6.98 |
| Σ^+^ | 369.51 | −3.78 | 381.85 | −3.8 | 371.49 | −3.79 | 378.83 | −3.8 |
| Σ^+^ | 570.3 | 0 | 580.70 | 0 | 574.91 | 0 | 581.97 | 0 |
| Σ^+^ | 1042.63 | −39.06 | 1055.90 | −39.85 | 1042.47 | −39.09 | 1048.12 | −39.47 |

^a^ with frozen core of metals.  ^b^ without frozen core of metals.

**Table S23.** Calculated harmonic vibrational wavenumbers$\nu$ in cm^−1^ and ^16/18^O isotopic shifts $\Delta\nu$ in cm^−1^ for triplet OCaF_2_ (^3^A) species at CCSD(T)/awCVTZ-PP level (no frozen core of metal).

| Symmetry | $\nu$(^16^O) | ∆$\nu$(^16/18^O) |
| --- | --- | --- |
| A | 25.71 | −0.08 |
| A | 55.24 | −0.63 |
| A | 135.53 | −4.14 |
| A | 207.10 | −6.91 |
| A | 481.04 | −0.11 |
| A | 586.31 | −0.13 |

**Table S24.** Calculated harmonic vibrational wavenumbers$\nu$ in cm^−1^and ^16/18^O isotopic shifts $\Delta\nu$ in cm^−1^ for side-on FSr(η^2^-OF) species at CCSD(T) level.

| **side-on FSr(η^2^-OF) (^1^A)** | | | | | | | | |
| --- | --- | --- | --- | --- | --- | --- | --- | --- |
| Symmetry | aVTZ-PP^a^ | | aVTZ-PP^b^ | | awCVTZ-PP^a^ | | awCVTZ-PP^b^ | |
|  | $\nu$(^16^O) | ∆$\nu$(^16/18^O) | $\nu$(^16^O) | ∆$\nu$(^16/18^O) | $\nu$(^16^O) | ∆$\nu$(^16/18^O) | $\nu$(^16^O) | ∆$\nu$(^16/18^O) |
| A | 42.80 | −0.18 | 49.30 | −0.17 | 19.00 | 0 | 19.51 | −0.01 |
| A | 78.01 | −1.52 | 77.39 | −1.68 | 62.86 | −0.42 | 57.57 | −0.76 |
| A | 277.30 | −1.11 | 304.08 | −0.89 | 320.87 | −1.05 | 280.65 | −0.81 |
| A | 426.17 | −20.07 | 416.39 | −19.55 | 437.99 | −17.55 | 403.17 | −19.14 |
| A | 489.98 | −0.18 | 491.50 | −0.04 | 589.77 | −1.07 | 477.38 | −0.10 |
| A | 804.38 | −23.63 | 787.03 | −23.36 | 791.82 | −23.44 | 790.10 | −23.49 |

^a^ with frozen core of metals.  ^b^ without frozen core of metals.

**Table S25.** Calculated harmonic vibrational wavenumbers$\nu$ in cm^−1^ and ^16/18^O isotopic shifts $\Delta\nu$ in cm^−1^ for end-on FSrOF species at CCSD(T) level.

| **end-on FSrOF (^1^A')** | | | | | | | | |
| --- | --- | --- | --- | --- | --- | --- | --- | --- |
| Symmetry | aVTZ-PP^a^ | | aVTZ-PP^b^ | | awCVTZ-PP^a^ | | awCVTZ-PP^b^ | |
|  | $\nu$(^16^O) | ∆$\nu$(^16/18^O) | $\nu$(^16^O) | ∆$\nu$(^16/18^O) | $\nu$(^16^O) | ∆$\nu$(^16/18^O) | $\nu$(^16^O) | ∆$\nu$(^16/18^O) |
| A' | 32.19 | −0.01 | 47.74 | −0.02 | 18.31 | 0 | 16.23 | 0 |
| A'' | 163.43 | −7.07 | 171.5 | −7.43 | 164.76 | −7.12 | 172.85 | −7.47 |
| A' | 177.10 | −7.93 | 179.64 | −7.72 | 165.99 | −7.50 | 174.43 | −7.53 |
| A' | 334.50 | −4.76 | 352.73 | −4.76 | 327.59 | −4.76 | 334.01 | −4.81 |
| A' | 484.10 | 0 | 487.56 | −0.01 | 480.55 | 0 | 477.23 | 0 |
| A' | 1007.44 | −37.23 | 1021.26 | −38.18 | 998.14 | −36.69 | 1003.02 | −36.99 |

^a^ with frozen core of metals.  ^b^ without frozen core of metals.

**Table S26.** Calculated harmonic vibrational wavenumbers$\nu$ in cm^−1^ and ^16/18^O isotopic shifts $\Delta\nu$ in cm^−1^ for triplet OSrF_2_ (^3^A) species at CCSD(T)/awCVTZ-PP level (no frozen core of metal).

| Symmetry | $\nu$(^16^O) | ∆$\nu$(^16/18^O) |
| --- | --- | --- |
| A | 44.33 | −0.74 |
| A | 81.22 | −0.21 |
| A | 155.45 | −5.56 |
| A | 177.96 | −6.27 |
| A | 445.32 | −0.27 |
| A | 475.69 | −0.02 |

**Table S27.** Calculated harmonic vibrational wavenumbers$\nu$ in cm^−1^, IR intensities in km mol^−1^ (in parentheses) and ^16/18^O isotopic shifts $\Delta\nu$ in cm^−1^ for side-on FBa(η^2^-OF) species at CCSD(T) level.

| **side-on FBa(η^2^-OF) (^1^A)** | | | | | | | | | |
| --- | --- | --- | --- | --- | --- | --- | --- | --- | --- |
| Symmetry | aVTZ-PP^a^ | | aVTZ-PP^b^ | | awCVTZ-PP^a^ | | awCVTZ-PP^b^ | | |
|  | $\nu$(^16^O) | ∆$\nu$(^16/18^O) | $\nu$(^16^O) | ∆$\nu$(^16/18^O) | $\nu$(^16^O) | ∆$\nu$(^16/18^O) | $\nu$(^16^O) | ∆$\nu$(^16/18^O) |  |
| A | 39.75 | −1.35 | 46.48 | −0.26 | 36.93 | −0.20 | 40.22 | −0.25 |  |
| A | 75.96 | −2.01 | 79.78 | −1.83 | 73.56 | −1.52 | 75.25 | −1.61 |  |
| A | 252.79 | −0.88 | 259.60 | −0.71 | 248.92 | −0.82 | 252.05 | −0.68 |  |
| A | 365.98 | −18.30 | 371.36 | −18.55 | 361.95 | −18.07 | 364.55 | −18.23 |  |
| A | 443.11 | −0.32 | 444.38 | −0.13 | 436.81 | −0.28 | 433.17 | −0.17 |  |
| A | 803.07 | −23.77 | 799.78 | −23.75 | 803.85 | −23.80 | 800.22 | −23.78 |  |

^a^ with frozen core of metals.  ^b^ without frozen core of metals.

**Table S28.** Calculated harmonic vibrational wavenumbers$\nu$ in cm^−1^, IR intensities in km mol^−1^ (in parentheses) and ^16/18^O isotopic shifts $\Delta\nu$in cm^−1^ for end-on FBaOF species at CCSD(T) level.

| **end-on FBaOF (^1^A')** | | | | | | | | |
| --- | --- | --- | --- | --- | --- | --- | --- | --- |
| Symmetry | aVTZ-PP^a^ | | aVTZ-PP^b^ | | awCVTZ-PP^a^ | | awCVTZ-PP^b^ | |
|  | $\nu$(^16^O) | ∆$\nu$(^16/18^O) | $\nu$(^16^O) | ∆$\nu$(^16/18^O) | $\nu$(^16^O) | ∆$\nu$(^16/18^O) | $\nu$(^16^O) | ∆$\nu$(^16/18^O) |
| A' | 45.49 | −0.01 | 50.59 | −0.03 | 44.47 | −0.01 | 50.73 | −0.01 |
| A'' | 135.36 | −5.77 | 143.28 | −6.16 | 134.17 | −5.67 | 141.6 | −6.05 |
| A' | 143.77 | −6.83 | 159.04 | −6.86 | 138.52 | −6.52 | 154.92 | −6.61 |
| A' | 304.47 | −5.59 | 313.65 | −5.46 | 302.96 | −5.66 | 305.16 | −5.56 |
| A' | 440.45 | −0.03 | 440.34 | −0.03 | 436.47 | −0.03 | 431.15 | −0.04 |
| A' | 968.42 | −34.81 | 971.79 | −35.19 | 964.54 | −34.56 | 963.45 | −34.53 |

^a^ with frozen core of metals.  ^b^ without frozen core of metals.

**Table S29.** Calculated harmonic vibrational wavenumbers$\nu$ in cm^−1^ and ^16/18^O isotopic shifts $\Delta\nu$ in cm^−1^ for triplet OBaF_2_ (^3^A) species at CCSD(T)/awCVTZ-PP level (no frozen core of metal).

| Symmetry | $\nu$(^16^O) | $\nu$(^18^O) |
| --- | --- | --- |
| A | −17.4 | −16.65 |
| A | 82.06 | 81.95 |
| A | 128.70 | 122.69 |
| A | 146.79 | 143.26 |
| A | 400.40 | 400.12 |
| A | 429.39 | 429.37 |


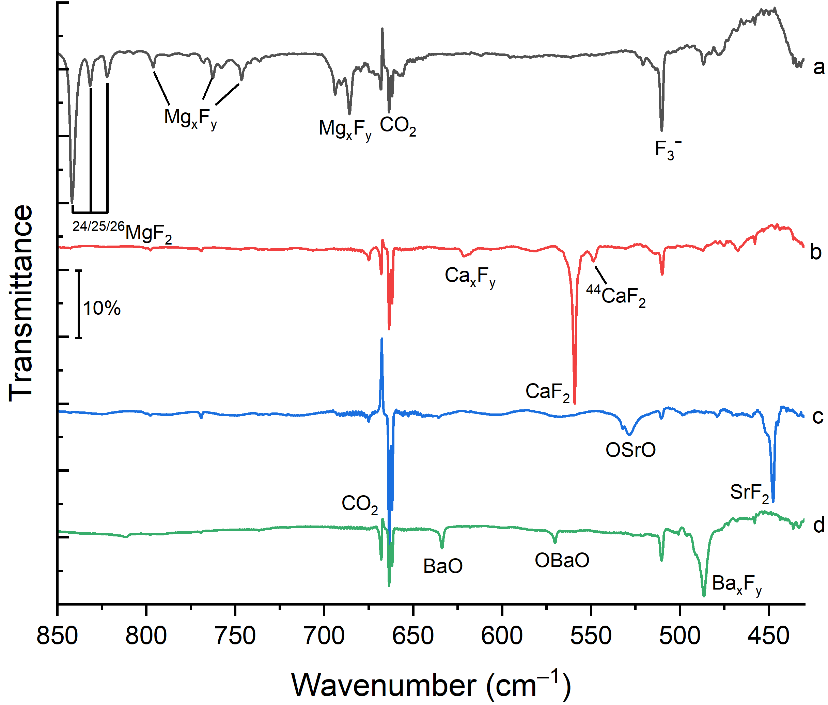
IR spectra

**Figure S1.** IR spectra of argon matrices: (a) after co-deposition of laser-ablated Mg atoms with 0.5% F_2_; (b) after co-deposition of laser-ablated Ca atoms with 0.5% F_2_; (c) after co-deposition of laser-ablated Sr atoms with 0.5% F_2_; (d) after co-deposition of laser-ablated Ba atoms with 0.5% F_2_.


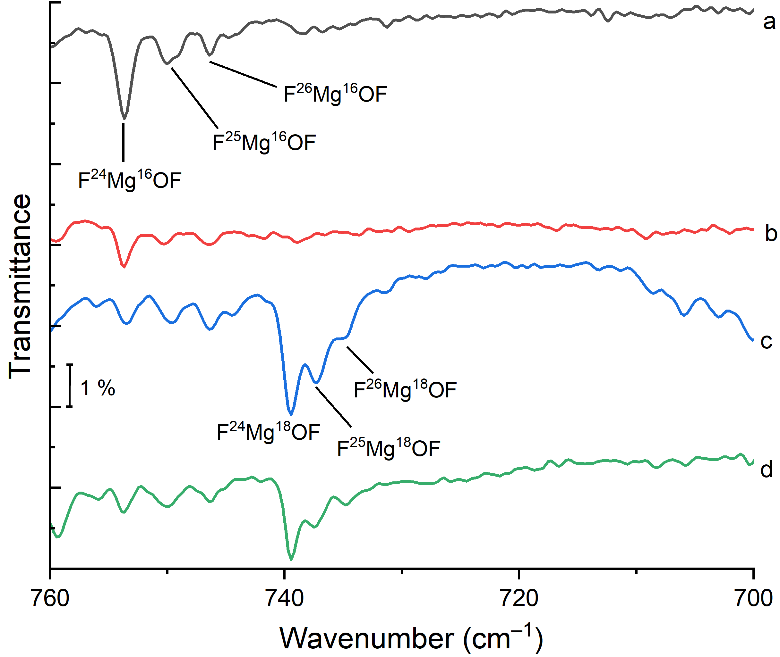


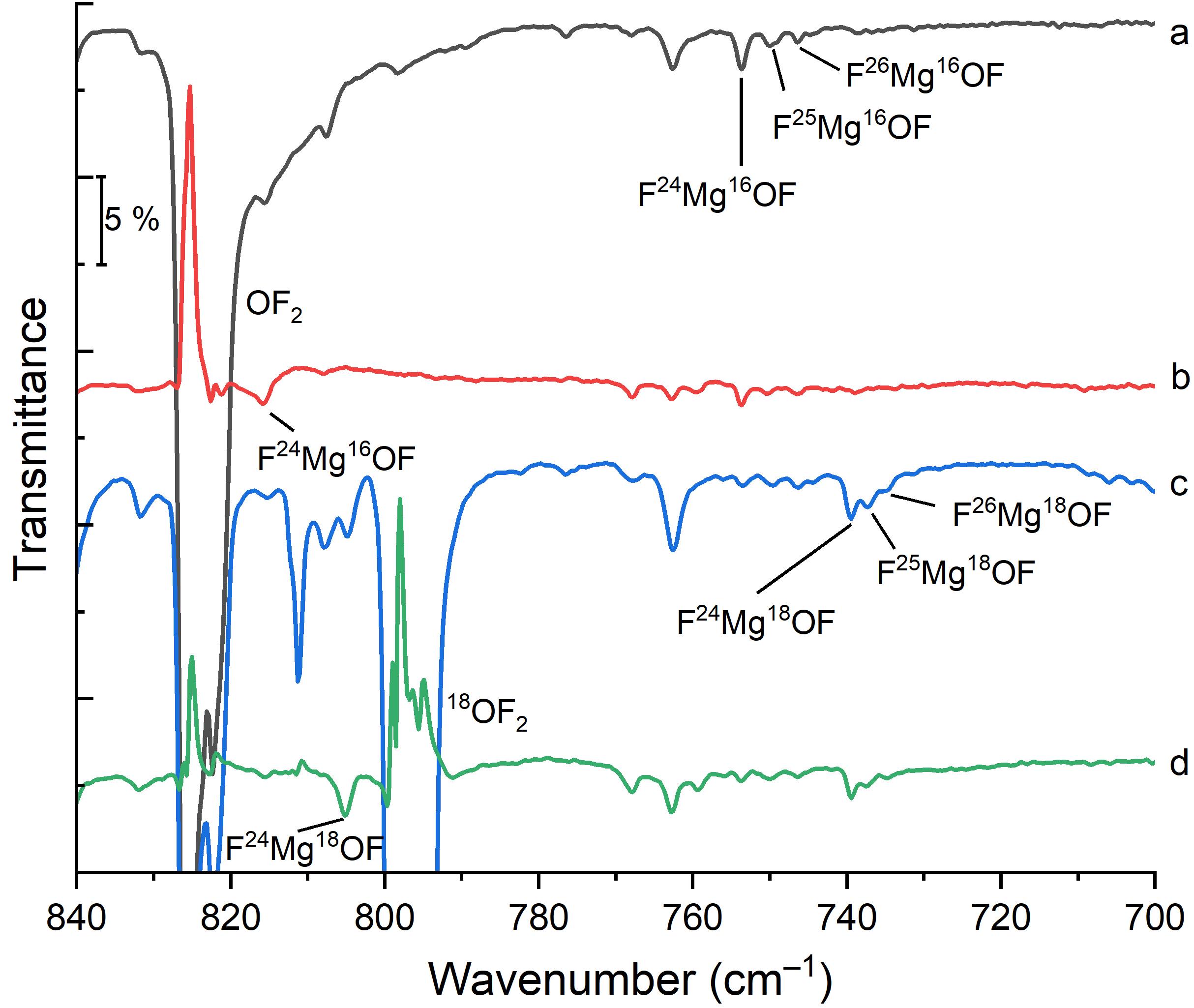
**Figure S2.** IR spectra of argon matrices in the region of 760−700 cm^−1^: (a) IR spectrum of reaction products of laser-ablated Mg atoms with 0.5% OF_2_ at 10 K; (b) difference spectrum after irradiation with 273 nm for 10 min; (c) IR spectrum of reaction products of laser-ablated Mg atoms with 0.5% ^18^OF_2_ at 10 K; (d) difference spectrum after irradiation with 273 nm for 10 min.

**Figure S3.** IR spectra of argon matrices in the region of 840−700 cm^−1^: (a) IR spectrum of reaction products of laser-ablated Mg atoms with 0.5% OF_2_ at 10 K; (b) difference spectrum after irradiation with 273 nm for 10 min; (c) IR spectrum of reaction products of laser-ablated Mg atoms with 0.5% ^18^OF_2_ at 10 K; (d) difference spectrum after irradiation with 273 nm for 10 min.


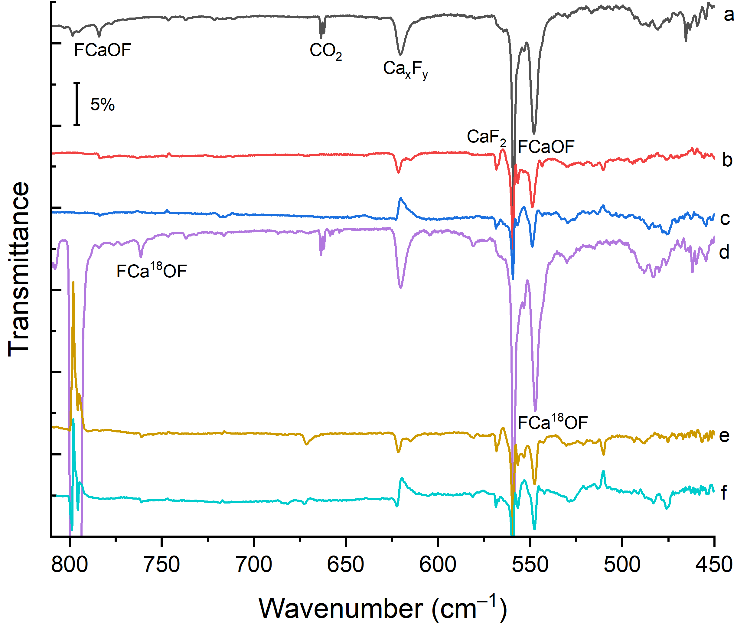


**Figure S4.** IR spectra of argon matrices: (a) IR spectrum of reaction products of laser-ablated Ca atoms with 0.5% OF_2_ at 10 K; (b) difference spectrum after irradiation with 455 nm for 10 min; (c) difference spectrum after full arc irradiation (λ > 220 nm) for 10 min; (d) IR spectrum of reaction products of laser-ablated Ca atoms with 0.5% ^18^OF_2_ at 10 K; (e) difference spectrum after irradiation with 455 nm for 10 min; (f) difference spectrum after full arc irradiation (*λ* > 220 nm) for 10 min.

**
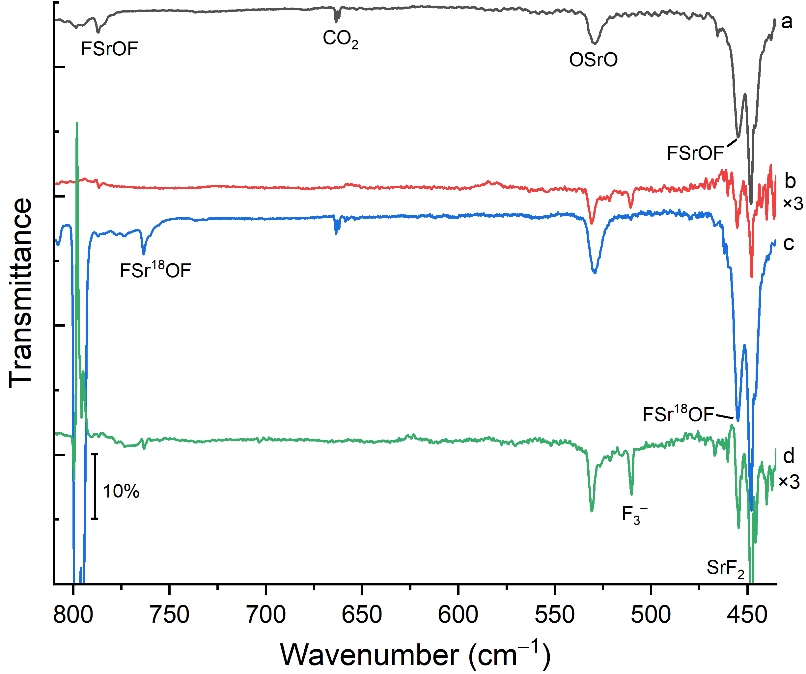
**

**Figure S5.** IR spectra of argon matrices: (a) IR spectrum of reaction products of laser-ablated Sr atoms with 0.5% OF_2_ at 10 K; (b) difference spectrum after irradiation with 455 nm for 10 min; (c) IR spectrum of reaction products of laser-ablated Sr atoms with 0.5% ^18^OF_2_ at 10 K; (d) difference spectrum after irradiation with 455 nm for 10 min.


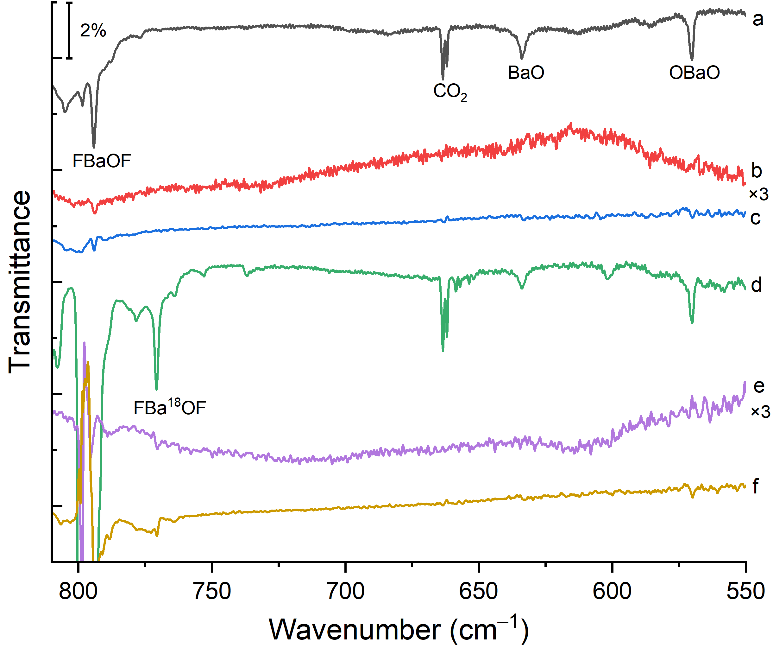


**Figure S6.** IR spectra of argon matrices in the region of 830−550 cm^−1^: (a) IR spectrum of reaction products of laser-ablated Ba atoms with 0.5% OF_2_ at 10 K; (b) difference spectrum after irradiation with 656 nm for 10 min; (c) difference spectrum after annealing to 15 K; (d) IR spectrum of reaction products of laser-ablated Ba atoms with 0.5% ^18^OF_2_ at 10 K; (e) difference spectrum after irradiation with 656 nm for 10 min; (f) difference spectrum after annealing to 15 K.


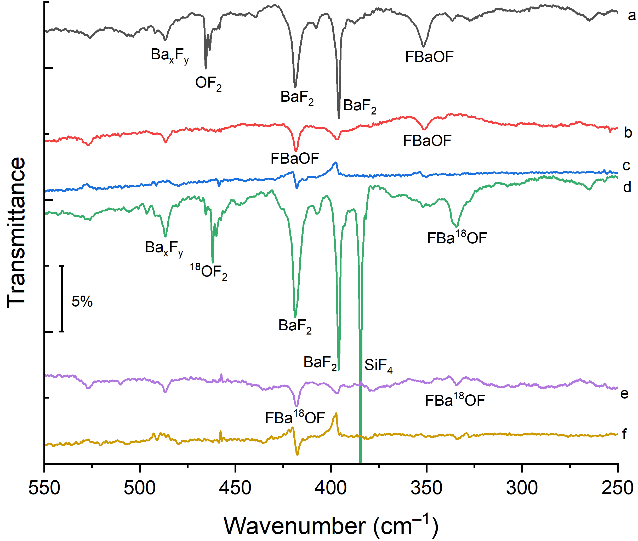


**Figure S7.** Far-IR spectra of argon matrices in the region of 550−250 cm^−1^: (a) IR spectrum of reaction products of laser-ablated Ba atoms with 0.5% OF_2_ at 10 K; (b) difference spectrum after irradiation with 656 nm for 10 min; (c) difference spectrum after annealing to 15 K; (d) IR spectrum of reaction products of laser-ablated Ba atoms with 0.5% ^18^OF_2_ at 10 K; (e) difference spectrum after irradiation with 656 nm for 10 min; (f) difference spectrum after annealing to 15 K.

**Table S30.** Calculated harmonic vibrational wavenumbers $\nu$ in cm^−1^, IR intensities in km mol^−1^ (in parentheses) and ^16/18^O isotopic shifts $\Delta\nu$ in cm^−1^ for OMF (M = Mg, Ca, Sr, Ba) species.

| species | B3LYP-D3^a^ | | CCSD(T)^b^ | |
| --- | --- | --- | --- | --- |
|  | $\nu$(^16^O) | ∆$\nu$^16/18^O) | $\nu$(^16^O) | ∆$\nu$(^16/18^O) |
| O^24^MgF | 147.9 (124) | −1.7 | 145.0 | −1.7 |
|  | 154.9 (119) | −1.8 | 151.1 | −1.8 |
|  | 541.5 (0) | −18.0 | 550.9 | −18.4 |
|  | 834.0 (126) | −7.1 | 845.2 | −7.0 |
| O^25^MgF | 146.1 (121) | −1.8 | 143.3 | −1.7 |
|  | 153.1 (116) | −1.8 | 149.3 | −1.8 |
|  | 541.5 (0) | −18.1 | 550.9 | −18.5 |
|  | 824.0 (123) | −7.2 | 835.2 | −7.1 |
| O^26^MgF | 144.5 (119) | −1.8 | 141.7 | −1.7 |
|  | 151.3 (113) | −1.9 | 147.6 | −1.8 |
|  | 541.5 (0) | −18.1 | 550.9 | -18.5 |
|  | 814.7 (121) | −7.2 | 825.8 | -7.1 |
| OCaF | 89.4 (71) | −1.7 | 90.3 | −2.0 |
|  | 473.6 (55) | −18.2 | 462.6 | −18.4 |
|  | 577.8 (240) | −2.2 | 583.5 | −2.9 |
| OSrF | 79.1 (53) | −1.8 | 38.1 | −0.8 |
|  | 376.8 (80) | −18.3 | 463.6 | −16.1 |
|  | 477.0 (149) | −0.2 | 486.1 | −7.5 |
| OBaF | 74.8 (40) | −2.0 | 67.4 | −2.2 |
|  | 363.8 (98) | −18.6 | 415.3 | −18.2 |
|  | 429.9 (124) | −0.2 | 428.7 | −3.5 |

´

^a^B3LYP-D3/def2-TZVP. ^b^ CCSD(T)/awCVTZ(-PP) without frozen core of metals.

Bonding analysis data

**Table S31.** NPA charges and Wiberg bond orders of F'M(η^2^-OF) (M = Mg, Ca, Sr, Ba) at the B3LYP-D3/def2-TZVP level of theory. All values are in atomic units.

| Property | Atom / Bond | F'Mg(η^2^-OF) | F'Ca(η^2^-OF) | F'Sr(η^2^-OF) | F'Ba(η^2^-OF) |
| --- | --- | --- | --- | --- | --- |
| NPA Charge | F' | −0.91 | −0.91 | −0.91 | −0.91 |
|  | M | 1.83 | 1.82 | 1.84 | 1.82 |
|  | O | −0.58 | −0.57 | −0.58 | −0.59 |
|  | F | −0.34 | −0.34 | −0.34 | −0.33 |
| Wiberg bond orders | F'−M | 0.17 | 0.18 | 0.17 | 0.19 |
|  | M−O | 0.12 | 0.13 | 0.12 | 0.13 |
|  | O−F | 0.88 | 0.88 | 0.88 | 0.89 |
|  | M−F | 0.05 | 0.04 | 0.04 | 0.03 |
| Mayer bond orders | F'−M | 0.79 | 0.63 | 0.54 | 0.59 |
|  | M−O | 0.54 | 0.37 | 0.33 | 0.37 |
|  | O−F | 0.73 | 0.74 | 0.74 | 0.76 |
|  | M−F | 0.27 | 0.17 | 0.15 | 0.14 |

**Table S 32.** Properties of selected bond critical points for F'M(η^2^-OF) calculated at B3LYP-D3/def2-TZVP level. All units are in atomic unit.

| bond | Electron density  *ρ* | Laplacian of electron density ∇^2^*ρ* | Energy density H | ELF | Potential energy density V(r) | Lagrangian kinetic energy G(r) | \|V(r)\|/G |
| --- | --- | --- | --- | --- | --- | --- | --- |
| Mg-F' | 0.083 | 0.762 | 0.010 | 0.059 | −0.169 | 0.179 | 0.944 |
| Mg-F | Not detectable due to too low electron density | | | | | | |
| Mg-O | 0.063 | 0.481 | 0.007 | 0.060 | −0.105 | 0.113 | 0.929 |
| Ca-F' | 0.084 | 0.495 | −0.004 | 0.116 | −0.131 | 0.128 | 1.023 |
| Ca-F | 0.041 | 0.263 | 0.006 | 0.050 | −0.054 | 0.060 | 0.850 |
| Ca-O | 0.061 | 0.298 | −0.002 | 0.113 | −0.079 | 0.077 | 1.026 |
| Sr-F' | 0.076 | 0.379 | −0.007 | 0.129 | −0.109 | 0.101 | 1.079 |
| Sr-F | 0.035 | 0.209 | 0.005 | 0.050 | −0.042 | 0.047 | 0.893 |
| Sr-O | 0.055 | 0.227 | −0.003 | 0.125 | −0.063 | 0.060 | 1.021 |
| Ba-F' | 0.078 | 0.559 | −0.014 | 0.185 | −0.099 | 0.085 | 1.165 |
| Ba-F | 0.032 | 0.175 | 0.004 | 0.054 | −0.034 | 0.039 | 0.872 |
| Ba-O | 0.054 | 0.184 | -0.005 | 0.166 | −0.055 | 0.050 | 1.100 |

ETS-NOCV analysis


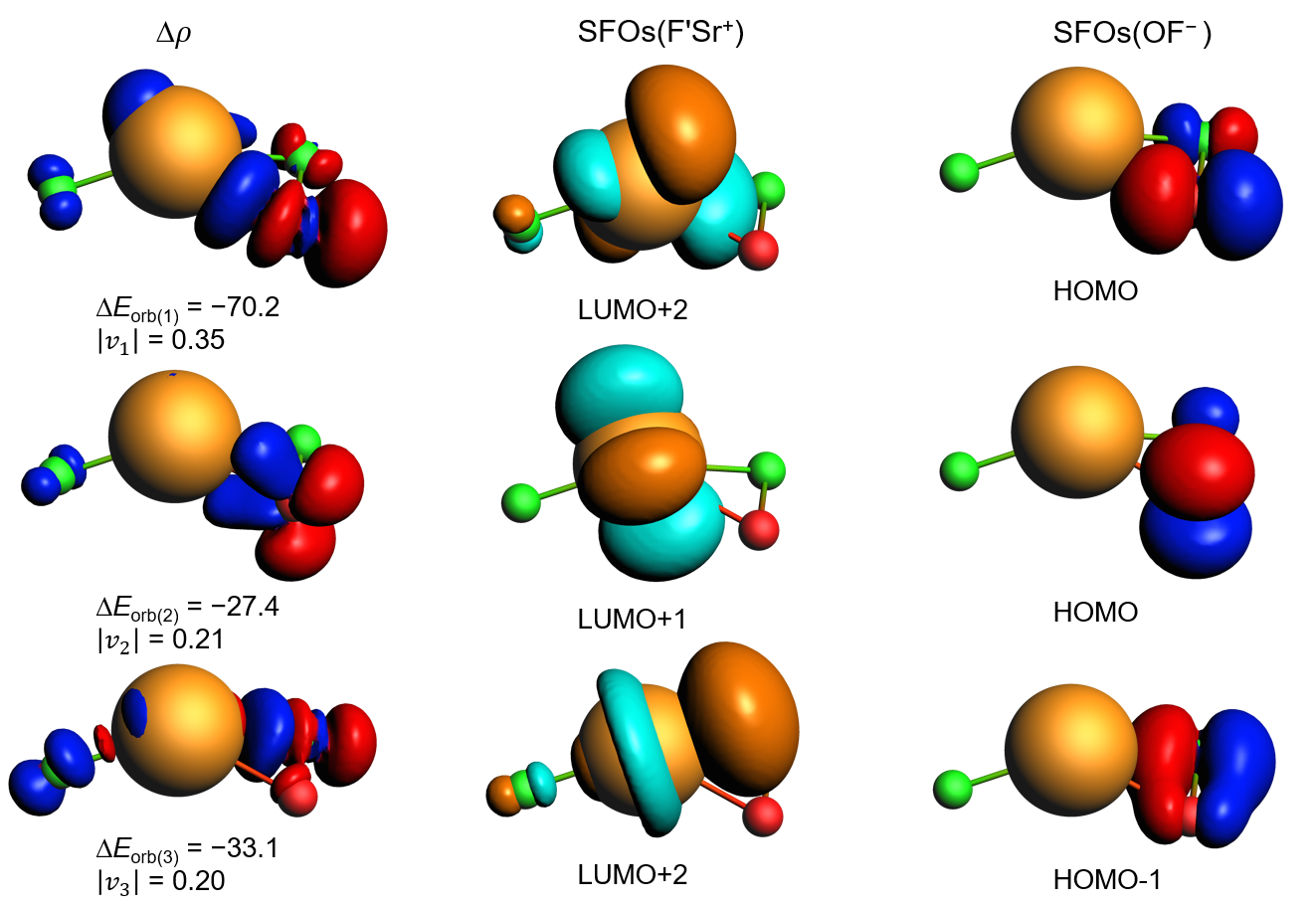


**Figure S8.** Deformation densities ∆*ρ* (the electronic charge flow is red to blue), corresponding eigenvalues * * and Δ*E*_orb_ interaction energiesin kJ mol^−1^, as well as shape of the most relevant respective symmetrized fragment orbitals (SFOs) of the fragments F'M^+^ and OF^−^ from ETS-NOCV calculations for F'Ba(η^2^-OF) performed at B3LYP-D3(BJ)/TZ2P//B3LYP-D3/def2-TZVP level. The isosurface values for ∆*ρ* is 0.001 au, and 0.05 au for the SFOs.


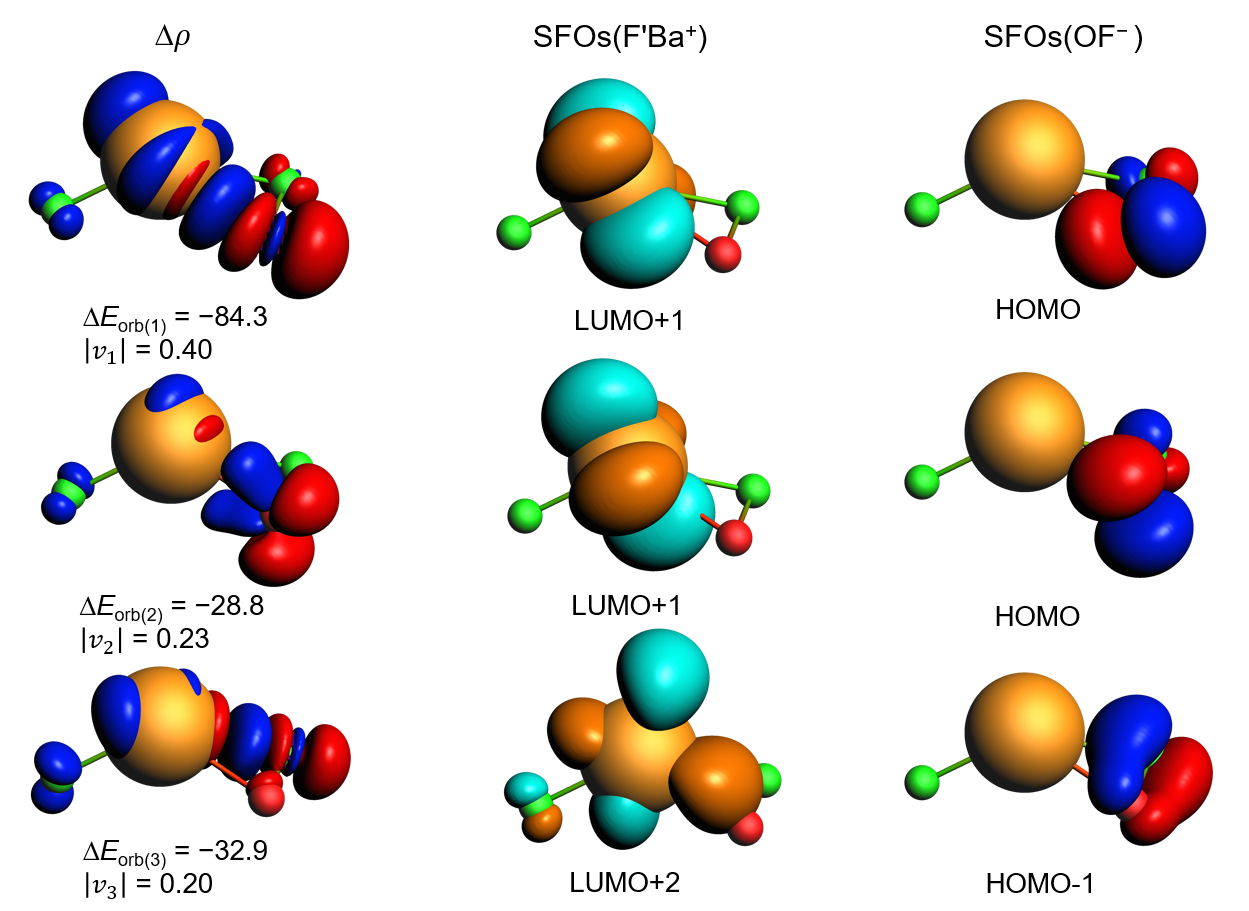


**Figure S9.** Deformation densities ∆*ρ* (the electronic charge flow is red to blue), corresponding eigenvalues * * and Δ*E*_orb_ interaction energiesin kJ mol^−1^, as well as shape of the most relevant respective symmetrized fragment orbitals (SFOs) of the fragments F'M^+^ and OF^−^ from ETS-NOCV calculations for F'Ba(η^2^-OF) performed at B3LYP-D3(BJ)/TZ2P//B3LYP-D3/def2-TZVP level. The isosurface values for ∆*ρ* is 0.001 au, and 0.05 au for the SFOs.

Calculated molecular structures

Calculated atomic coordinates (in Å) of species for optimized structures.

**B3LYP-D3/def2-TZVP**

**OMgF**

Mg 0.00000000 0.00000000 0.03069800

O 0.00000000 0.00000000 1.89285300

F 0.00000000 0.00000000 -1.72346700

**FMg(η^2^-OF) (side-on) (^1^A')**

Mg 0.00000000 0.40167700 0.00000000

F -0.73277100 1.99019900 0.00000000

O 1.16217400 -1.09615900 0.00000000

F -0.30027200 -1.55140500 0.00000000

**FMgOF (end-on) (^1^∑^+^)**

Mg 0.00000000 0.00000000 0.70233000

F 0.00000000 0.00000000 2.44936300

O 0.00000000 0.00000000 -1.05511000

F 0.00000000 0.00000000 -2.44792800

**OMgF_2_ (^3^A'')**

Mg 0.00000000 0.33079800 0.00000000

O -0.59470300 -1.67305300 0.00000000

F -0.88765800 1.84205100 0.00000000

F 1.41628400 -0.79595700 0.00000000

**OCaF**

Ca 0.00000000 0.33840100 0.00000000

O 2.02829700 -0.26408300 0.00000000

F -1.80293000 -0.51726100 0.00000000

**FCa(η^2^-OF)(side-on) (^1^A')**

Ca 0.00000000 0.39693500 0.00000000

O -0.40505200 -1.74516300 0.00000000

F 1.09959800 -1.58501900 0.00000000

F -0.73955200 2.25419800 0.00000000

**FCaOF (end-on) (^1^∑^+^)**

Ca 0.00000000 0.00000000 0.63283000

O 0.00000000 0.00000000 -1.38953500

F 0.00000000 0.00000000 -2.80342300

F 0.00000000 0.00000000 2.63227700

**OCaF_2_ (^3^A)**

Ca 0.34109100 -0.00969100 0.19850000

O -1.79952500 1.08282800 -0.10781700

F -1.45267900 -1.04038600 -0.12517900

F 2.29427700 0.09940700 -0.22009400

**OSrF**

Sr 0.00000000 0.29759300 0.00000000

O 2.07392200 -0.55422300 0.00000000

F -1.84348600 -0.76386200 0.00000000

**FSr(η^2^-OF) (side-on) (^1^A)**

Sr -0.30519700 -0.18497300 -0.18259700

O 1.73297600 0.94055200 -0.12610100

F 2.02220200 -0.42185800 0.45785200

F -2.27401400 0.36680700 0.42520300

**FSrOF (end-on) (^1^A')**

Sr 0.00000000 0.56629100 0.00000000

O -0.85075300 -1.43054300 0.00000000

F -1.03647700 -2.84168500 0.00000000

F 1.79270200 1.72227500 0.00000000

**OSrF_2_ (^3^A)**

Sr -0.28073400 -0.01452400 -0.28794500

F -2.20295900 0.12817100 0.62747200

O 1.99978600 1.07792400 0.27011900

F 1.61069100 -1.02500400 0.34819100

**OBaF**

Ba 0.00000000 0.21410400 0.00000000

O 2.16473100 -0.42865500 0.00000000

F -1.92420500 -0.95117600 0.00000000

**FBa(η^2^-OF) (side-on) (^1^A)**

Ba -0.27409500 -0.27059900 -0.07714100

O 1.85703900 0.87312900 -0.53535100

F 2.20051400 0.01028900 0.64495900

F -2.14573300 0.89732400 0.31089600

**FBaOF (end-on) (^1^A')**

Ba 0.00000000 0.51856100 0.00000000

O -1.08017300 -1.51438800 0.00000000

F -1.21475000 -2.93948200 0.00000000

F 2.17490400 1.05900200 0.00000000

**OBaF_2_ (^3^A)**

Ba -0.26284100 -0.20972100 -0.22010000

F -2.04168400 0.80849200 0.68252500

O 2.19283000 1.08870800 -0.37157200

F 1.72795500 -0.47130500 1.01727100

**CCSD(T)/aug-cc-pVTZ with frozen core**

**OMgF**

UCCSD(T)/AVTZ ENERGY=-374.55826744

Mg 0.0000000000 0.0000000000 0.0293860682

O 0.0000000000 0.0000000000 1.9050859439

F 0.0000000000 0.0000000000 -1.7343220121

**FMg(η^2^-OF) (side-on) (^1^A')**

CCSD(T)/AUG-CC-PVTZ ENERGY=-474.28470634

Mg -0.3840801368 0.1222379447 0.0000000000

O 1.4159678986 0.7688648746 0.0000000000

F -2.1340295851 -0.0767819677 0.0000000000

F 1.3855318234 -0.7710208516 0.0000000000

**FMgOF (end-on) (^1^∑^+^)**

CCSD(T)/AVTZ ENERGY=-474.25507542

Mg 0.0000000000 0.0000000000 0.7085780158

F 0.0000000000 0.0000000000 2.4666797195

O 0.0000000000 0.0000000000 -1.0618388856

F 0.0000000000 0.0000000000 -2.4647688497

**OMgF_2_ (^3^A'')**

UCCSD(T)/AVTZ ENERGY=-474.28298484

Mg -0.2681443400 0.3009641506 0.0000000000

O 1.1117256850 -1.5170961014 0.0000000000

F 1.0125014877 1.5246429741 0.0000000000

F -1.7901328328 -0.6048110233 0.0000000000

**CCSD(T)/aug-cc-pVTZ-(PP) with frozen core**

**FCa(η^2^-OF)(side-on) (^1^A')**

CCSD(T)/AUG-CC-PVTZ,CA=AUG-CC-PVTZ-PP ENERGY=-311.06490418

Ca -0.1247664340 0.3624651053 0.0000000000

O -0.3849354712 -1.8318523785 0.0000000000

F 1.1106904455 -1.4997121538 0.0000000000

F -0.6460475402 2.2901724271 0.0000000000

**FCaOF(end-on) (^1^∑^+^)**

CCSD(T)/AUG-CC-PVTZ,CA=AUG-CC-PVTZ-PP ENERGY=-311.07481864

Ca 0.0000000000 0.0000000000 0.6379855019

O 0.0000000000 0.0000000000 -1.3944927970

F 0.0000000000 0.0000000000 -2.8244886250

F 0.0000000000 0.0000000000 2.6532159201

**FSr(η^2^-OF)(side-on) (^1^A)**

CCSD(T)/AUG-CC-PVTZ,SR=AUG-CC-PVTZ-PP ENERGY=-304.87002984

Sr -0.2873359447 -0.2028238160 -0.2608983760

F -2.2025017523 0.3729502208 0.4666602741

O 1.6975251349 0.9457728657 -0.1332121206

F 1.9682825621 -0.4153692705 0.5018002224

**FSrOF(end-on) (^1^A)**

CCSD(T)/AUG-CC-PVTZ,SR=AUG-CC-PVTZ-PP ENERGY=-304.90286524

Sr 0.1148254798 0.4974024801 0.0000000000

F -1.1901803373 -2.8645111880 0.0000000000

O -0.7643354037 -1.4902466985 0.0000000000

F 1.7455102613 1.8764554063 0.0000000000

**FBa(η^2^-OF)(side-on) (^1^A)**

CCSD(T)/AUG-CC-PVTZ,BA=AUG-CC-PVTZ-PP ENERGY=-299.66832030

F -2.1472810603 0.8777874097 0.3265371353

O 1.8775890864 0.8661478599 -0.5619899065

F 2.1675453642 0.0066990619 0.6659144414

Ba -0.2601233904 -0.2404943314 -0.0870916702

**FBaOF(end-on) (^1^A')**

CCSD(T)/AUG-CC-PVTZ,BA=AUG-CC-PVTZ-PP ENERGY=-299.69147195

O 1.0222343202 1.5714364336 0.0000000000

F 1.3728553448 2.9757354273 0.0000000000

F -2.1916272574 -1.2140519435 0.0000000000

Ba -0.0834424076 -0.4568099174 0.0000000000

**CCSD(T)/aug-cc-pVTZ-(PP) without frozen core**

**FCa(η^2^-OF)(side-on) (^1^A')**

CCSD(T)/AUG-CC-PVTZ,CA=AUG-CC-PVTZ-PP ENERGY=-311.33774213

Ca -0.1241644738 0.3552878755 0.0000000000

O -0.3833281852 -1.8197324442 0.0000000000

F 1.1094682619 -1.5011978395 0.0000000000

F -0.6470346028 2.2867154083 0.0000000000

**FCaOF(end-on) (^1^∑^+^)**

CCSD(T)/AUG-CC-PVTZ,CA=AUG-CC-PVTZ-PP ENERGY=-311.32156689

Ca 0.0000000000 0.0000000000 0.6277152652

O 0.0000000000 0.0000000000 -1.3777217673

F 0.0000000000 0.0000000000 -2.8005870934

F 0.0000000000 0.0000000000 2.6228135955

**FSr(η^2^-OF)(side-on) (^1^A)**

CCSD(T)/AUG-CC-PVTZ,SR=AUG-CC-PVTZ-PP ENERGY=-305.15785542

Sr -0.2855970316 -0.1668658896 -0.3035779145

F -2.1939682450 0.3365594347 0.4987847626

O 1.7462841593 0.9377955414 -0.1588996959

F 1.9092511173 -0.4069590865 0.5380428476

**FSrOF(end-on) (^1^A')**

CCSD(T)/AUG-CC-PVTZ,SR=AUG-CC-PVTZ-PP ENERGY=-305.14378784

Sr -0.0248452155 0.5699040364 0.0000000000

F -1.0857684632 -2.8325221866 0.0000000000

O -0.7534843319 -1.4407396263 0.0000000000

F 1.7699180106 1.7224577764 0.0000000000

**FBa(η^2^-OF)(side-on) (^1^A)**

CCSD(T)/AUG-CC-PVTZ,BA=AUG-CC-PVTZ-PP ENERGY=-299.99124059

Ba -0.2594520175 -0.2473552603 -0.1272110305

O 1.8920826871 0.8472879782 -0.5692883414

F 2.1335575184 0.0339345172 0.6846840277

F -2.1284581881 0.8762727649 0.3551853443

**FBaOF(end-on) (^1^A')**

CCSD(T)/AUG-CC-PVTZ,BA=AUG-CC-PVTZ-PP ENERGY=-299.98039293

Ba -0.0476900981 -0.4660777789 0.0000000000

O 0.9800702817 1.5604006171 0.0000000000

F 1.3595650660 2.9503101950 0.0000000000

F -2.1719252496 -1.1683230332 0.0000000000

**CCSD(T)/aug-cc-pwCVTZ-(PP) with frozen core**

**FCa(η^2^-OF)(side-on) (^1^A')**

CCSD(T)/AUG-CC-PVTZ,CA=AUG-CC-PWCVTZ-PP ENERGY=-311.09020113

Ca -0.1016254713 0.3654518819 0.0000000000

O -0.3915002923 -1.8131217282 0.0000000000

F 1.1084816295 -1.5086460021 0.0000000000

F -0.6604148658 2.2773888484 0.0000000000

**FCaOF(end-on) (^1^∑^+^)**

CCSD(T)/AUG-CC-PVTZ,CA=AUG-CC-PWCVTZ-PP ENERGY=-311.08594987

Ca 0.0000000000 0.0000000000 0.6382579154

O 0.0000000000 0.0000000000 -1.3909725457

F 0.0000000000 0.0000000000 -2.8208348403

F 0.0000000000 0.0000000000 2.6457694705

**FSr(η^2^-OF)(side-on) (^1^A)**

CCSD(T)/AUG-CC-PVTZ,SR=AUG-CC-PWCVTZ-PP ENERGY=-304.93217150

Sr -0.1177404499 -0.1213927912 0.2686177672

F 0.2891844562 0.2425979926 2.3283207424

O -0.0065312230 0.9752344050 -1.7759642656

F 0.2593307992 -0.5040267543 -2.0715584291

**FSrOF(end-on) (^1^A')**

CCSD(T)/AUG-CC-PVTZ,SR=AUG-CC-PWCVTZ-PP ENERGY=-304.93768723

Sr 0.2612607303 0.4048602792 0.0000000000

F -1.2918464921 -2.8554965753 0.0000000000

O -0.7435750378 -1.5237128487 0.0000000000

F 1.6799807997 1.9934491448 0.0000000000

**FBa(η^2^-OF)(side-on) (^1^A)**

CCSD(T)/AUG-CC-PVTZ,BA=AUG-CC-PWCVTZ-PP ENERGY=-299.69703407

F -2.1552844540 0.8740248212 0.3288169950

O 1.8847765584 0.8619867304 -0.5638308879

F 2.1708632538 0.0073660142 0.6675675439

Ba -0.2626253583 -0.2332375657 -0.0891836510

**FBaOF(end-on) (^1^A')**

CCSD(T)/AUG-CC-PVTZ,BA=AUG-CC-PWCVTZ-PP ENERGY=-299.70718176

O 1.0289355081 1.5673130689 0.0000000000

F 1.3681323251 2.9750555874 0.0000000000

F -2.1902355040 -1.2149412801 0.0000000000

Ba -0.0868123292 -0.4511173762 0.0000000000

**CCSD(T)/aug-cc-pwCVTZ-(PP) without frozen core**

**OMgF**

UCCSD(T)/AUG-CC-PVTZ,MG=AUG-CC-PWCVTZ ENERGY=-374.81242709

Mg 0.0000000000 0.0000000000 0.0295709773

O 0.0000000000 0.0000000000 1.8851637757

F 0.0000000000 0.0000000000 -1.7145847531

**FMg(η^2^-OF) (side-on) (^1^A')**

CCSD(T)/AUG-CC-PVTZ,MG=AUG-CC-PWCVTZ ENERGY=-474.54030209

Mg -0.3758190038 0.1173047039 0.0000000000

O 1.3975961410 0.7689879442 0.0000000000

F -2.1071030437 -0.0736913868 0.0000000000

F 1.3687159065 -0.7693012614 0.0000000000

**FMgOF (end-on) (^1^∑^+^)**

CCSD(T)/AUG-CC-PVTZ,MG=AUG-CC-PWCVTZ ENERGY=-474.51091352

Mg 0.0000000000 0.0000000000 0.7017736491

F 0.0000000000 0.0000000000 2.4396933949

O 0.0000000000 0.0000000000 -1.0465469919

F 0.0000000000 0.0000000000 -2.4462700520

**OMgF_2_ (^3^A'')**

UCCSD(T)/AUG-CC-PVTZ,MG=AUG-CC-PWCVTZ ENERGY=-474.53752691

Mg -0.2619637992 0.2926962525 0.0000000000

O 1.0939015742 -1.4929956683 0.0000000000

F 1.0029878126 1.5047473008 0.0000000000

F -1.7689755876 -0.6007478850 0.0000000000

**OCaF**

UCCSD(T)/AUG-CC-PVTZ,CA=AUG-CC-PWCVTZ-PP ENERGY=-211.68945569

Ca 0.0000000000 -0.2488392973 0.0490154641

O 0.0000000000 0.3013834840 2.1122682812

F 0.0000000000 0.2711556399 -1.8822405726

**FCa(η^2^-OF)(side-on) (^1^A')**

CCSD(T)/aug-cc-pVTZ,CA=AUG-CC-PWCVTZ-PP energy= -311.427358724798

Ca -0.0952051952 0.3628537435 0.0000000000

O -0.3903439032 -1.8037027485 0.0000000000

F 1.1083839725 -1.5173993533 0.0000000000

F -0.6678938740 2.2793213583 0.0000000000

**FCaOF(end-on) (^1^∑^+^)**

CCSD(T)/aug-cc-pVTZ,CA=AUG-CC-PWCVTZ-PP energy= -311.409708507049

Ca 0.0000000000 0.0000000000 0.6330215453

O 0.0000000000 0.0000000000 -1.3826026914

F 0.0000000000 0.0000000000 -2.8067343288

F 0.0000000000 0.0000000000 2.6285354748

**OCaF_2_ (^3^A)**

UCCSD(T)/AUG-CC-PVTZ,CA=AUG-CC-PWCVTZ-PP ENERGY=-311.41727582

Ca 0.3405381829 -0.0815188039 -0.0597726436

O -1.8685999015 1.1697913987 -0.0882362935

F -1.4277879138 -1.1052833052 -0.0197133964

F 2.3386296324 0.1296907105 -0.1053576666

**OSrF**

UCCSD(T)/AUG-CC-PVTZ,SR=AUG-CC-PWCVTZ-PP ENERGY=-205.52543484

Sr 0.0000000000 -0.1141599228 0.0522464079

O 0.0000000000 0.3130062794 2.1534049031

F 0.0000000000 0.2629052436 -2.0544367157

**FSr(η^2^-OF)(side-on) (^1^A)**

CCSD(T)/AUG-CC-PVTZ,SR=AUG-CC-PWCVTZ-PP ENERGY=-305.26092263

Sr -0.1179646205 -0.1207285149 0.2694093663

F 0.2894577957 0.2430712966 2.3345228987

O -0.0067042264 0.9683311823 -1.7869043039

F 0.2602370207 -0.5017501678 -2.0721983212

**FSrOF(end-on) (^1^A')**

CCSD(T)/AUG-CC-PVTZ,SR=AUG-CC-PWCVTZ-PP ENERGY=-305.24620916

Sr 0.2551637390 0.3968271462 0.0000000000

F -1.2870706463 -2.8470870514 0.0000000000

O -0.7415550553 -1.5208352907 0.0000000000

F 1.6792819627 1.9901951959 0.0000000000

**OSrF_2_ (^3^A)**

CCSD(T)/AUG-CC-PVTZ,SR=AUG-CC-PWCVTZ-PP ENERGY=-305.24975423

Sr -0.2831119623 -0.0801005724 -0.2492584762

O 2.0653265989 1.1739652405 0.2770772235

F 1.5732222069 -1.0687095667 0.3177319767

F -2.2283068434 0.1403348986 0.6121592759

**OBaF**

UCCSD(T)/AUG-CC-PVTZ,BA=AUG-CC-PWCVTZ-PP ENERGY=-200.31130944

Ba 0.0000000000 -0.1632018535 0.0429481572

O 0.0000000000 0.7112028905 2.1026804500

F 0.0000000000 0.5807693951 -2.0812115637

**FBa(η^2^-OF)(side-on) (^1^A)**

CCSD(T)/AUG-CC-PVTZ,BA=AUG-CC-PWCVTZ-PP ENERGY=-300.04131899

F -2.1640245929 0.8731404748 0.3313407174

O 1.8938358296 0.8568135828 -0.5609104237

F 2.1710711132 0.0113587308 0.6637654788

Ba -0.2631523500 -0.2311727882 -0.0908257725

**FBaOF(end-on) (^1^A')**

CCSD(T)/AUG-CC-PVTZ,BA=AUG-CC-PWCVTZ-PP ENERGY=-300.02985324

Ba -0.0685797409 -0.4515337402 0.0000000000

O 1.0075463583 1.5633048488 0.0000000000

F 1.3638376200 2.9611053443 0.0000000000

F -2.1827842375 -1.1965664529 0.0000000000

**OBaF_2_ (^3^A)**

CCSD(T)/AUG-CC-PVTZ,BA=AUG-CC-PWCVTZ-PP ENERGY=-300.03015398

F -2.0681474657 0.8296201088 0.6496924778

O 2.2872891431 1.1775337256 -0.4657575181

F 1.6687252511 -0.5040017152 1.0369969174

Ba -0.2718869285 -0.2572421192 -0.1461918771

# References

[26] M. J. Frisch, G. W. Trucks, H. B. Schlegel, G. E. Scuseria, M. A. Robb, J. R. Cheeseman, G. Scalmani, V. Barone, G. A. Petersson, H. Nakatsuji, X. Li, M. Caricato, A. V. Marenich, J. Bloino, B. G. Janesko, R. Gomperts, B. Mennucci, H. P. Hratchian, J. V. Ortiz, A. F. Izmaylov, J. L. Sonnenberg, D. Williams-Young, F. Ding, F. Lipparini, F. Egidi, J. Goings, B. Peng, A. Petrone, T. Henderson, D. Ranasinghe, V. G. Zakrzewski, J. Gao, N. Rega, G. Zheng, W. Liang, M. Hada, M. Ehara, K. Toyota, R. Fukuda, J. Hasegawa, M. Ishida, T. Nakajima, Y. Honda, O. Kitao, H. Nakai, T. Vreven, K. Throssell, J. A. Montgomery, Jr., J. E. Peralta, F. Ogliaro, M. J. Bearpark, J. J. Heyd, E. N. Brothers, K. N. Kudin, V. N. Staroverov, T. A. Keith, R. Kobayashi, J. Normand, K. Raghavachari, A. P. Rendell, J. C. Burant, S. S. Iyengar, J. Tomasi, M. Cossi, J. M. Millam, M. Klene, C. Adamo, R. Cammi, J. W. Ochterski, R. L. Martin, K. Morokuma, O. Farkas, J. B. Foresman, and D. J. Fox, *Gaussian16*, Gaussian, Inc., Wallingford CT, **2016**.

[27] A. D. Becke, *J. Chem. Phys.* **1992**, *96*, 2155.

[28] C. Lee, W. Yang, R. G. Parr, *Phys. Rev. B* **1988**, *37*, 785.

[29] P. J. Stephens, F. J. Devlin, C. F. Chabalowski, M. J. Frisch, *J. Phys. Chem.* **1994**, *98*, 11623.

[30] S. H. Vosko, L. Wilk, M. Nusair, *Can. J. Phys.* **1980**, *58*, 1200.

[31] S. Grimme, J. Antony, S. Ehrlich, H. Krieg, *J. Chem. Phys.* **2010**, *132*, 154104.

[32] F. Weigend, R. Ahlrichs, *Phys. Chem. Chem. Phys.* **2005**, *7*, 3297.

[33] E. D. Glendening, J. K. Badenhoop, A. E. Reed, J. E. Carpenter, J. A. Bohmann, C. M. Morales, P. Karafiloglou, C. R. Landis, F. Weinhold, *NBO 7.0*, Theoretical Chemistry Institute, University of Wisconsin, Madison, WI, **2018**.

[34] Tian Lu, Feiwu Chen, *J. Comput. Chem.* **2012**, *33*, 580.

[35] M. Mitoraj, A. Michalak, *J. Mol. Model.* **2007**, *13*, 347.

[36] M. P. Mitoraj, A. Michalak, T. Ziegler, *J. Chem. Theory Comput.* **2009**, *5*, 962.

[37] T. Ziegler, A. Rauk, *Theoret. Chim. Acta* **1977**, *46*, 1.

[38] G. te Velde, F. M. Bickelhaupt, E. J. Baerends, C. Fonseca Guerra, van Gisbergen, S. J. A., J. G. Snijders, T. Ziegler, *J. Comput. Chem.* **2001**, *22*, 931.

[39] H.-J. Werner, P. J. Knowles, P. Celani, W. Györffy, A. Hesselmann, D. Kats, G. Knizia, A. Köhn, T. Korona, D. Kreplin, R. Lindh, Q. Ma, F. R. Manby, A. Mitrushenkov, G. Rauhut, M. Schütz, K. R. Shamasundar, T. B. Adler, R. D. Amos, J. Baker, S. J. Bennie, A. Bernhardsson, A. Berning, J. A. Black, P. J. Bygrave, R. Cimiraglia, D. L. Cooper, D. Coughtrie, M. J. O. Deegan, A. J. Dobbyn, K. Doll and M. Dornbach, F. Eckert, S. Erfort, E. Goll, C. Hampel, G. Hetzer, J. G. Hill, M. Hodges and T. Hrenar, G. Jansen, C. Köppl, C. Kollmar, S. J. R. Lee, Y. Liu, A. W. Lloyd, R. A. Mata, A. J. May, B. Mussard, S. J. McNicholas, W. Meyer, T. F. Miller III, M. E. Mura, A. Nicklass, D. P. O'Neill, P. Palmieri, D. Peng, K. A. Peterson, K. Pflüger, R. Pitzer, I. Polyak, P. Pulay, M. Reiher, J. O. Richardson, J. B. Robinson, B. Schröder, M. Schwilk and T. Shiozaki, M. Sibaev, H. Stoll, A. J. Stone, R. Tarroni, T. Thorsteinsson, J. Toulouse, M. Wang, M. Welborn and B. Ziegler, *MOLPRO, version 2021.3, a package of ab initio programs*, **2021**.

[40] H.-J. Werner, P. J. Knowles, F. R. Manby, J. A. Black, K. Doll, A. Heßelmann, D. Kats, A. Köhn, T. Korona, D. A. Kreplin et al., *J. Chem. Phys.* **2020**, *152*, 144107.

[41] H.‐J. Werner, P. J. Knowles, G. Knizia, F. R. Manby, M. Schütz, *WIREs Comput Mol Sci* **2012**, *2*, 242.

[42] T. H. Dunning, *J. Chem. Phys.* **1989**, *90*, 1007.

[43] R. A. Kendall, T. H. Dunning, R. J. Harrison, *J. Chem. Phys.* **1992**, *96*, 6796.

[44] B. P. Prascher, D. E. Woon, K. A. Peterson, T. H. Dunning, A. K. Wilson, *Theoret. Chim. Acta* **2011**, *128*, 69.

[45] I. S. Lim, H. Stoll, P. Schwerdtfeger, *J. Chem. Phys.* **2006**, *124*, 34107.

[46] J. G. Hill, K. A. Peterson, *J. Chem. Phys.* **2017**, *147*, 244106.

# Author Contributions

Xiya Xia did all the experiments and calculations, wrote the initial draft of the manuscript. Robert Medel and Sebastian Riedel managed the project and revised the manuscript.
